# Ipomoea carnea mitigates ethanol-induced ulcers in irradiated rats via Nrf2/HO−1 pathway: an in vivo and in silico study

**DOI:** 10.1038/s41598-024-53336-1

**Published:** 2024-02-12

**Authors:** Mosad A. Ghareeb, Hala Sh. Mohammed, Tarek Aboushousha, Dina M. Lotfy, Maha A. M. El-Shazly, Mansour Sobeh, Eman F. S. Taha

**Affiliations:** 1 Medicinal Chemistry Department, Theodor Bilharz Research Institute, Kornaish El-Nile, Warrak El-Hadar, Imbaba, P.O. Box 30, Giza, 12411 Egypt; 2 Department of Pharmacognosy and Medicinal Plants, Faculty of Pharmacy (Girls), Al-Azhar University, Cairo, 11311 Egypt; 3 Department of Pathology, Theodor Bilharz Research Institute, Kornaish El-Nile, Warrak El-Hadar, Imbaba, P.O. Box 30, Giza, 12411 Egypt; 4 Drug Radiation Research Department, National Center for Radiation Research and Technology, (NCRRT), Egyptian Atomic Energy Authority (EAEA), Cairo, Egypt; 5 AgroBioSciences, Mohammed VI Polytechnic University, Lot 660, Hay Moulay Rachid, 43150 Ben-Guerir, Morocco; 6 Health Radiation Research Department, National Center for Radiation Research and Technology, (NCRRT), Egyptian Atomic Energy Authority, Cairo, Egypt

**Keywords:** Biochemistry, Biological techniques, Plant sciences

## Abstract

The aim of this study was to investigate the potential of *Ipomoea carnea* flower methanolic extract (ICME) as a natural gastroprotective therapy against ethanol-induced gastric ulcers, particularly in individuals exposed to ionizing radiation (IR). The study focused on the Nrf2/HO−1 signaling pathway, which plays a crucial role in protecting the gastrointestinal mucosa from oxidative stress and inflammation. Male Wistar rats were divided into nine groups, the control group received distilled water orally for one week, while other groups were treated with ethanol to induce stomach ulcers, IR exposure, omeprazole, and different doses of ICME in combination with ethanol and/or IR. The study conducted comprehensive analyses, including LC-HRESI-MS/MS, to characterize the phenolic contents of ICME. Additionally, the Nrf2/HO−1 pathway, oxidative stress parameters, gastric pH, and histopathological changes were examined. The results showed that rats treated with IR and/or ethanol exhibited histopathological alterations, increased lipid peroxidation, decreased antioxidant enzyme activity, and reduced expression levels of Nrf2 and HO−1. However, pretreatment with ICME significantly improved these parameters. Phytochemical analysis identified 39 compounds in ICME, with flavonoids, hydroxybenzoic acids, and fatty acids as the predominant compounds. Virtual screening and molecular dynamics simulations suggested that ICME may protect against gastric ulceration by inhibiting oxidative stress and inflammatory mediators. In conclusion, this study demonstrates the potential of ICME as a natural gastroprotective therapy for preventing gastric ulcers. These findings contribute to the development of novel interventions for gastrointestinal disorders using natural plant extracts particularly in individuals with a history of radiation exposure**.**

## Introduction

Gastric ulcers (GU) are a prevalent gastrointestinal disorder characterized by the erosion of the stomach lining, leading to severe pain and discomfort^1^_._ GU affects 5–10% of the general population, with an annual incidence of 0.1–0.3%. Ulcers manifest as mucosal breaks in the gastrointestinal tract, primarily caused by excessive acid-peptic juice exposure^2^. Oxygen-derived free radicals are also involved in the pathogenesis of acute gastric mucosal injury and the delayed healing of chronic gastric ulcers^3^. The pathogenesis of GU is due to an imbalance between defensive factors and offensive agents, such as gastric acid, pepsin, and reactive oxygen species (ROS). The most crucial factors that cause such an imbalance between the mucus and the acid include *H. pylori* infection, non-steroidal anti-inflammatory drugs (NSAIDs), misuse of alcohol and excessive smoking.

Ethanol-induced GU in rats is characterized by the increased production of hydroperoxy free radicals and superoxide anions, leading to lipid peroxidation and oxidative stress in the gastric tissues^4^. In comparison to other GU models, ethanol-induced GU model offers distinct advantage due to close resemblence to the the symptoms observed in acute clinical peptic ulcer disease. It has been widely recognized that alcohol consumption leads to inflammation of the gastric mucosa. Upon admisnistration, ethanol rapidly penetrates the gastrointestinal mucosa, resulting in membrane disruption, erosion, and shedding of cells. These actions can lead to necrosis and the development of ulcers via increasing the permeability of the mucosa to stomach acid and promoting vasoactive substances production by the recruited macrophages, mast cells, and blood cells. Additionally, ethanol induces damage to the microvasculature via reducing cellular antioxidant levels, impairing blood flow, and escalating the production of pro-inflammatory cytokines and ROS^5^.

Gamma ionizing radiation (IR) is another factor that can cause gastric ulcer in rats. Exposure to gamma radiation can result in failure of the parietal cells to respond to the direct stimulation by histamine or pentagastrin, leading to a decrease in gastric acid secretion and gastric mucosal integrity^6^. Radiation therapy is a common treatment for various tumors, but its effectiveness is limited by its immediate and delayed side effects on normal tissues. The gastrointestinal tract, particularly the small intestine, is most sensitive to radiation effects. Radiation-induced lipid peroxidation and DNA damage are caused by the excessive generation of ROS, which interact with biological molecules, causing tissue damage^7^. IR can significantly impact gastric function, with studies showing an increase in gastric acid output and plasma gastrin levels in rats exposed to 2- or 6 Gray (Gy) whole-body gamma radiation^6^.

Most studies focused on either the effect of IR or ethanol separately for induction of GU in experimental animals. Both agents can damage the gastric mucosa by increasing ROS, decreasing antioxidants, activating nuclear factor kappa-light-chain-enhancer of activated B cells (NF-κB) pathway, and enhancing tumour necrosis factor alpha (TNF-α) and interleukin IL-6^8^.

Antacids, cell protection factors, proton pump inhibitors (like omeprazole, lansoprazole), and H_2_ receptor blockers (like cimetidine, ranitidine) are the most often recommended drugs for treating and preventing stomach ulcers. These drugs come at the risk of some adverse effects as they could cause gynecomastia, hypersensitivity, haematological abnormalities, impotence, and arrhythmia. It is therefore inevitable to use a natural anti-ulcer medication as an alternative with low adverse effects^9^.

The Nrf2 (nuclear factor erythroid 2-related factor 2)/HO−1 (heme oxygenase-1) signaling pathway has emerged as a key regulator of cellular defense mechanisms against oxidative stress and inflammation^10^. HO−1, an enzyme responsible for heme catabolism, plays a crucial role in the protection of gastrointestinal mucosa by exerting anti-inflammatory, antioxidant, and cytoprotective effects^11^. The Nrf2/HO−1 signaling pathway represents a novel and important mechanism for the protection against gastric damage induced by various insults, including NSAIDs, ethanol, and gamma irradiation. Activation of this pathway has been associated with the restoration of antioxidant levels, adaptive defense against oxidative stress, and the prevention of gastric mucosal damage. Therefore, the Nrf2/HO−1 pathway holds significant potential as a target for the development of novel therapeutic interventions for the management of gastric damage and ulceration^12^.

Taking altogether, there has been growing interest in the development of natural gastroprotective therapies to mitigate the harmful effects of GU^13^. Natural compounds derived from plants have shown promise due to their diverse pharmacological properties and minimal side effects^14^. *Ipomoea carnea* (*I. carnea*), commonly known as the pink morning glory, is a flowering plant widely distributed in the tropical and subtropical regions.

Several studies have reported the medicinal properties of *I. carnea*, including its anti-inflammatory, antioxidant, and cytoprotective effects due to their high contents of phenolic compounds that are well-known to furnish such activities^15^. *I. carnea* methanolic extract has been shown to have strong antioxidant properties^16^, while the plant's aqueous extract has been linked to beneficial effects against diabetes^17^. Furthermore, *I. carnea* leaves showed enhanced anti-inflammatory properties^18^. Because flavonoids and other chemicals with a free radical scavenging potential that are present in leaves, these plants have potent wound-healing effects^19^. The aerial portions of the plant, which include the stem, leaves, flowers, and seeds, are reported to contain a variety of metabolites, such as flavonoids, tannins, glycosides, alkaloids, carbohydrates, and phenolic compounds^20^.

By investigating the gastroprotective effects of *I. carnea* flower methanolic extract (ICME) in the context of gastric injury induced by gamma radiation alone or combined with ethanol, this study aimed to provide valuable insights into potential therapeutic interventions for the management of GU, particularly in individuals exposed to radiation. Furthermore, understanding the molecular mechanisms underlying the protective effects of ICME, including its modulation of the Nrf2/HO−1 signaling pathway, could pave the way for the development of novel drug targets and strategies for gastroprotection in the field of gastroenterology.

## Materials and methods

### Plant material

Flowers of *Ipomoea carnea* (*I. carnea*) were collected from Al Qaliobia Governorate, Egypt, in May 2020 (N 30° 10′ 43.95′′, E 31° 12′ 23.1696), in accordance with the relevant international guidelines and legislation, and with the necessary permissions obtained. The collection process adhered to standard practices for plant collection and preservation. Voucher specimens (No. I.c/fl/2020) were prepared from the freshly harvested samples and deposited in the herbarium of the Medicinal Chemistry Department at Theodor Bilharz Research Institute. The herbarium serves as a repository for authentic plant specimens and ensures their long-term preservation for future reference. Taxonomic identification and authentication of the collected plant material were performed by Mrs. Terese Labib, a taxonomy consultant at the Ministry of Agriculture and the former director of El-Orman Botanical Garden, Giza, Egypt. The identification process involved comparing the plant characteristics with established taxonomic references and expertise in plant taxonomy. To further validate the identification, the plant name was cross-verified on April 18th, 2012, using the plant verification website http://www.theplantlist.org. This online resource serves as a reliable reference for plant taxonomy and provides a comprehensive list of known plant species.

### Extraction

Fresh *I. carnea* flowers (1.7 kg) were extracted via maceration using methanol (4 × 3 L) at 25 °C. The mixed extracts were filtered and vacuum-evaporated at 40 °C to yield the dry extract (358 g, 21.06%). Subsequently, the methanol extract was stored for further chemical and biological investigations^21^.

### LC–ESI–MS/MS analysis

The chemical constituents of *I. carnea* flower parts extract was tentatively recognized using LC system SHIMADZU LC MS 8050 (Shimadzu, Japan, USA) coupled with a triple quadruple spectrometer with an ESI source according to the reported procedures^22^.

### Materials

Colorimetric assay kits for alanine aminotransferase (GPT; Cat. No.: AT 1034 (45), aspartate aminotransaminase (GOT; Cat. No.: AT 1034 (45)), urea (Cat. No.: UR 2110), creatinine (Cat. No.: CR 1251), catalase (CAT; Cat. No.: CA 2517), superoxide dismutase (SOD; Cat. No.: SD 2521), glutathione reduced (GSH; Cat. No.: GR 2511), glutathione peroxidase (GPx; Cat. No.: GP 2524) and malondialdehyde (MDA; Cat. No.: MD 2529) were purchased from the Bio Diagnostic company, Giza, Egypt. Rat nuclear factor erythroid 2-related protein 2 (Nrf2; Cat. No.: ER971) ELISA kit was obtained from Fine Test company, USA, and rat heme oxygenase-1 (HO−1; Cat. No.: E-EL-R0528) ELISA kit was acquired from Elabscience company, USA.

### Determination of phytochemical contents of ICME

Quantitative analyses were carried out on the extract from the plant material (ICME) to determine its chemical composition, specifically phytochemical components such as phenolics, flavonoids, alkaloids, and tannins. The total phenolic content of the ICME was measured using the Folin-Ciocalteu method, which has been previously described in a study^23^. Phenol contents was calculated in terms of mg catechol equivalent/g of dry sample (standard plot: y = 0.0966x, R^2^ = 0.9878). To assess the total flavonoid content, we employed the aluminum chloride colorimetric method, following the specified procedure^24^. Using the standard plot of quercetin (y = 0.0148x, R^2^ = 0.975), the flavonoid contents of *I. carnea* flower was calculated in terms of mg quercetin equivalent/g of dry sample. For the quantitative estimation of the total alkaloid content, we utilized the technique established by Sreevidya and Mehrotra using Dragendorff’s Reagent^25^. A calibration curve was constructed with the help of bismuth nitrate (y = 0.00017x-0.01723, R^2^ = 0.9), and the alkaloidal content was calculated in terms of mg per g of the extract^26^. Lastly, the total tannins content (TTC) was evaluated using a modified vanillin assay^27^. TTC amount of the test sample was calculated using the standard curve of tannic acid (y = 0.0014x + 0.023; R^2^ = 0.9987) in terms of mg tannic acid equivalent/g of dry weight of the extract^28^.

### LD_50_ of Ipomoea carnea methanolic extract (ICME)

A study of acute oral toxicity was conducted following the Organisation for Economic Cooperation and Development's (OECD) criteria^29^. By ingestion of the extracts into healthy albino male rats at dosages of 400, 800, 1600, and 3000 mg/kg body weight (b.wt.), the median lethal dose of the rats was estimated. Rats were checked 24 h later for any lethality or deaths (% of mortality). The LD_50_ of ICME was established using the procedure outlined by Chinedu et al. (2013)^30^. Before the experimentation, the rats were fasted for 12 h and then divided into five groups, each containing two animals. Group I represented the control, while groups II, III, and IV represented the ICAE (400, 800, 1600, and 3000 mg/kg b.wt.), respectively). Rats were then observed for 24 h, each group's mortality and toxicology symptoms were noted, and the LD_50_ was determined according to the next formula: LD_50_ = (M1 + M0)⁄2 = (1600 + 3000)⁄2 $=2300\mathrm{ mg}/{\text{kg}}$

Whereas: M0 is the upper dosage of ICAE that did not lead to fatality, and M1 is the minimal dosage of ICAE that lead to fatality. For biochemical studies, fixed dosage approaches have chosen doses of 300 and 500 mg/kg body weight.

### Experimental animals and ethical issues

The Egyptian organization for biological products and vaccines at Giza, Egypt, provided the male albino rats (150 ± 10 g). Before the investigation, the rats were acclimated for one week and given the usual feed (Rat pellets have 23% protein content, 6% fibers, 9% ash, and 4–5% fat content for a balanced rat diet) as well as unlimited access to water. Rats were kept in conventional circumstances that included humidity, temperature (20–22 °C), and a 12-h cycle of light and darkness. Before collecting samples, rats fasted all night. All experimental protocols and methodology were carried out following the Research Ethics Committee of the National Centre for Radiation Research and Technology (REC-NCRRT), Cairo, Egypt by applying the principles of replacement, reduction and refinement (the 3Rs) and gave its approval to the study. The protocol's serial number was 23A/22, 29/9/2022. This study was carried out in compliance with the Animal Research: Reporting of In Vivo Experiments (ARRIVE) guidelines.The Euthanasia of Animals was carried according to The American Veterinary Medical Association​ (AVMA) Guidelines for the Euthanasia of Animals (2020).

### Radiation processing

This process was achieved utilizing a gamma cell-40 (cesium-137), Atomic Energy of Canada Limited, Ottawa, Canada, situated at NCRRT, Cairo, Egypt. At the time of the study, rats were given only one single dose of 6 Gray (Gy) of whole-body gamma radiation, provided at a dosage rate of 0.33 Gy/min. Radiation injury to the stomach can result from the chosen gamma-ray dose^31^.

### Induction of gastric ulcer (GU)

A dosage of 5 mL/kg body weight of ethanol was administered orally to induce gastric ulcers in rats after an 24-h fasting period before receiving the tested samples meanwhile the animals were allowed to freely access water until 2 h before triggering the study inorder to ensurre the the stomach empty protocol^32^.

#### Experimental groups

**Figure 1:**
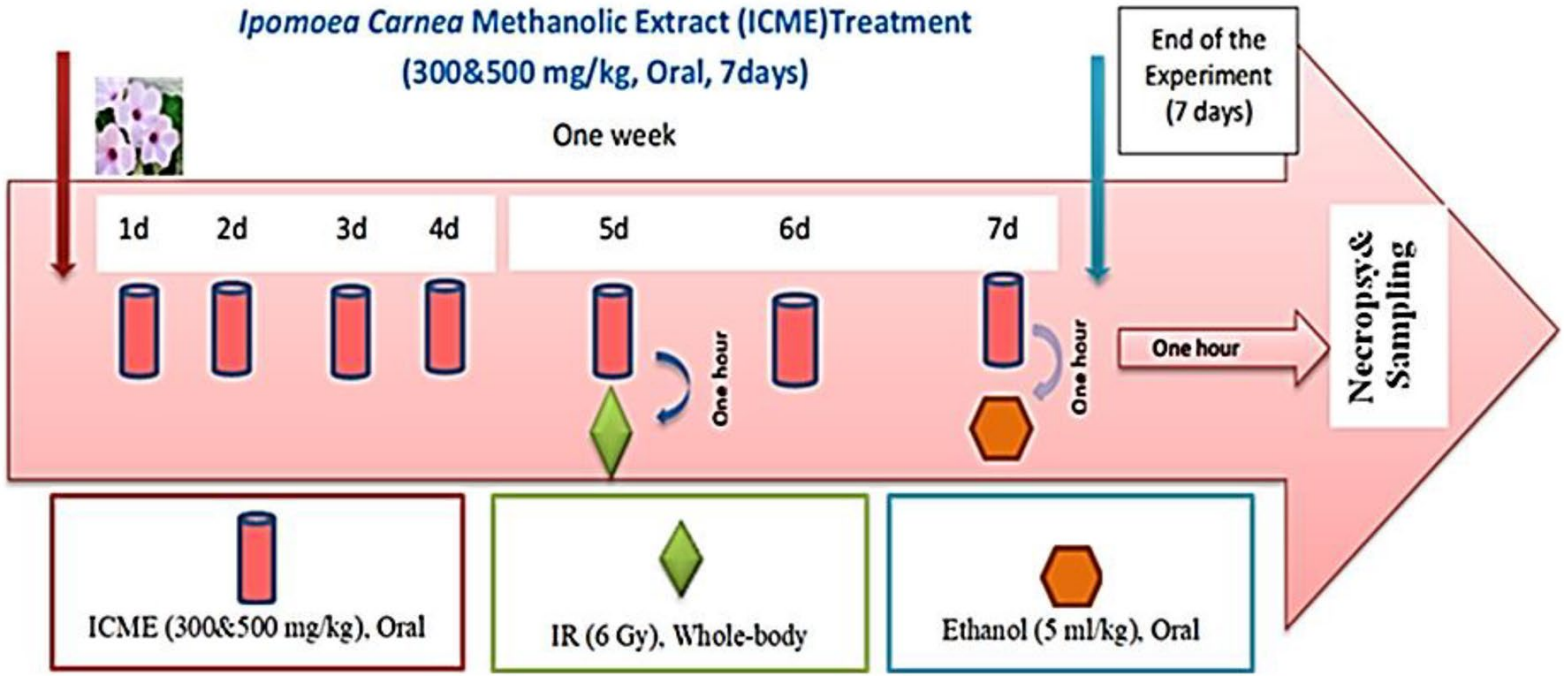
Experimental design.

**Figure 2:**
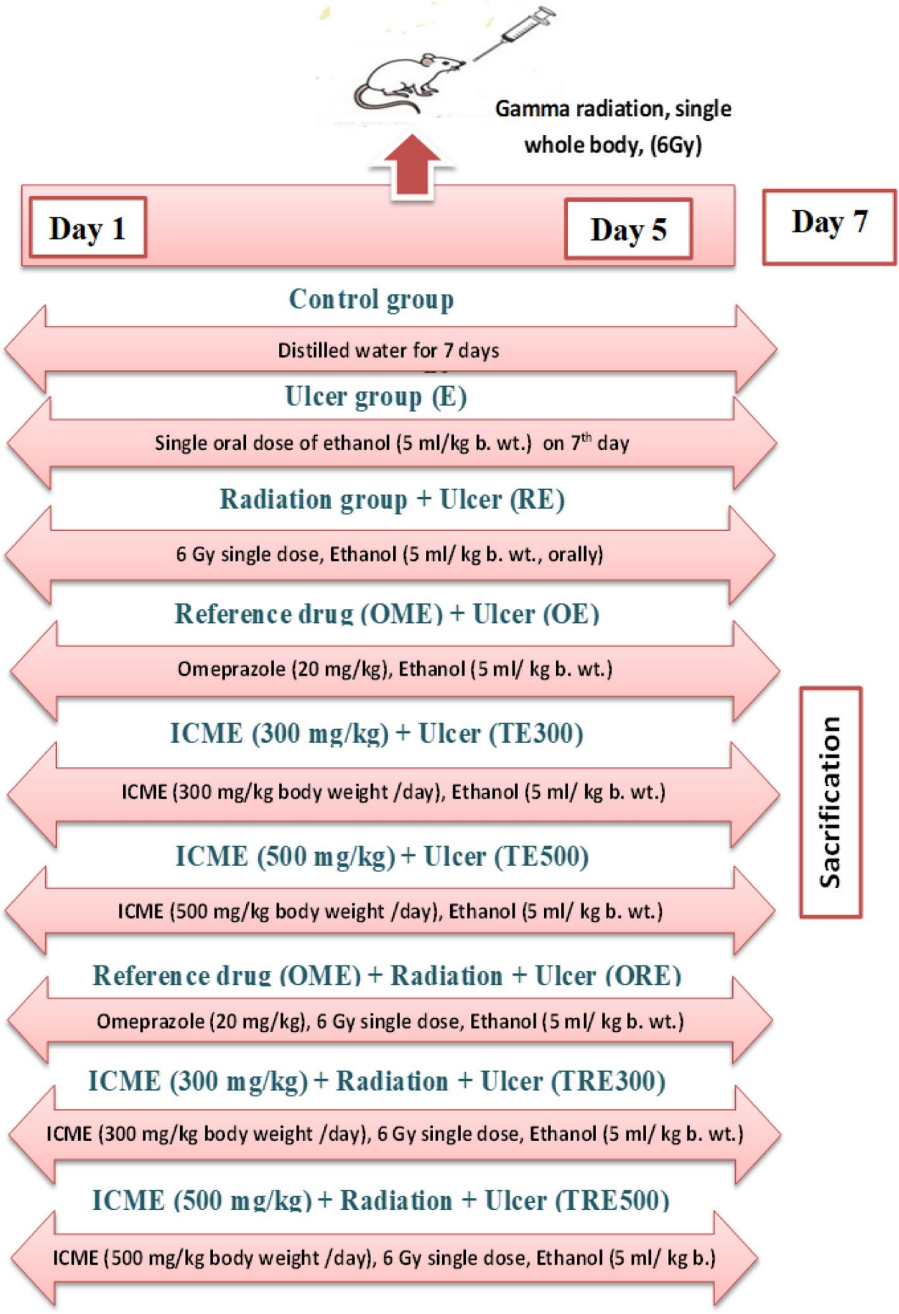
Experimental groups.

Fifty-four male albino rats were indiscriminately separated into nine groups of six rats each (Figs. 1, 2)**,** as follows:

- **Group 1 (Control group):** Rats were given orally (p.o.) distilled water (5 mL/kg. b.wt., p.o.) for a week.
- **Group 2 (Ethanol-induced ulcer group, E):** Rats were given orally distilled water for a week and received a single ethanol dosage (5 mL/kg b.wt., p.o.) on day 7, an hour after the last dose of treatment and an hour before decapitation.
- **Group 3 (RE):** Rats were given distilled water (p.o.) for a week and subjected to a single dose whole-body gamma radiation (6 Gy) on day 5, then received ethanol as in Group 2.
- **Group 4 (Standard Group, OE):** The reference drug Omeprazole (OME, 20 mg/kg b.wt., p.o.) was supplied to rats for a week, then a single ethanol dosage was given as in Group 2.
- **Group 5 (low concentration ICME group, TE300):** Rats were given ICME (300 mg/kg b.wt./day, p.o.) for a week, then a single ethanol dosage was given as in Group 2.
- **Group 6 (High concentration ICME group, TE500):** Rats were given ICME (500 mg/kg b.wt./day, p.o.) for a week, then a single dose of ethanol was given as in Group 2.
- **Group 7 (Standard group, ORE):** The reference drug OME (20 mg/kg b.wt., p.o.) was given to rats as in Group 4 and received IR and ethanol as in Group 3.
- **Group 8 (TRE300):** Rats were given ICME as in Group 5 and subjected to IR and ethanol as in Group 3.
- **Group 9 (TRE500):** Rats were given ICME as in Group 6 and received IR and ethanol as in Group 3.

### Anesthesia and euthanization of rats

The Euthanasia of Animals was carried according to The American Veterinary Medical Association (AVMA) Guidelines for the Euthanasia of Animals (2020). After one hour of ethanol administration, the rats were decapitated under deep urethane anesthesia (1.5 g/kg b.w., intra peritoneal (i.p.))^33^.

### Tissue collection and preparation

The stomachs of each rat were separated immediately after decapitation, cut along their outer curvature, and cleaned using phosphate-buffered saline. Gastric contents were collected to determine the pH of the stomach. The stomach tissues were then dried and photographed to identify the presence of ulcer lesions. Each stomach tissue was dissected into two identical pieces. One portion of each animal was immediately placed in 10% (v/v) formalin for histopathological evaluations, while the remaining portion was homogenized to assess its biochemical characteristics^34^.

### Gastric pH measurement

Each stomach was cut at the point of greatest curvature, the gastric contents were drained into a centrifuge tube, and the tubes were centrifuged at 1000 rpm for 10 min at 4 °C. One mL of supernatant was collected and diluted with one mL of distilled water^35^, and the pH of the solution was determined utilizing a pH meter (HANNA® benchtop pH meters, HI 110, USA)^36^.

### Serum biochemical evaluation

The efficacy of the enzymes aspartate transaminase (AST), alanine transaminase (ALT), urea and creatinine in serum was measured using commercially available kits.

### Biochemical analysis of tissue homogenate

#### Oxidative stress and antioxidant enzyme measurements

Gastric damage was assessed at a gross level before collecting and homogenizing the stomach tissue. Subsequently, the supernatant was collected after centrifuging at 10,000 g for 10 min. Commercial assay kits were used to quantify the concentrations of MDA, GSH, SOD, GPx, and CAT in the stomach tissues, following the manufacturer's guidelines.

#### Determination of Nrf2 and HO−1 levels

According to the manufacturer’s recommendations, homogenates of stomach samples were used to measure Nrf2 and HO−1 levels using ELISA kits.

### Relative stomach weight

All rats were properly dissected under anesthesia on the seventh day of the treatment, and the full weight of the stomach was estimated^34^. The relative stomach weights of each rat were then determined as follows**:** Relative stomach weight = absolute stomach weight (g)/body weight of rat on sacrifice day (g) *100.

### Histological analysis

Small pieces of gastric glandular epithelium (1–2 cm) were sectioned and fixed immediately for 24 h in 10% formalin. Gastric tissue was dehydrated with ethanol and then cleared of xylene and paraffin using a tissue processing machine. For sliding, a 5 cm slice of paraffin-embedded tissue was used (Leica Rotation Microtome). Hematoxylin and eosin stains were applied to the prepared slides and examined under a compound microscope^37^. The scale bars were added using the ImageJ program.

### Statistical analysis

With the help of the statistical program GraphPad Prism 5.01 for Windows (GraphPad Software, San Diego, California, USA; www.graphpad.com), results were subjected to statistical analysis and tests of significance. The results were shown as the mean ± SEM (n = 6). The differences between groups were evaluated utilizing a one-way analysis of variance (ANOVA) and a post hoc Tukey test at a *p*-value < 0.05.

### Significance

The innovation of this study lies in the unique combination of gamma radiation-induced gastric mucosal injury followed by exposure to ethanol for the induction of gastric ulcers, and the evaluation of a gastric protective extract in this context. This approach simulates a scenario where individuals who have undergone radiation therapy or have been exposed to radiation are subsequently exposed to factors that can further damage the gastric mucosa, such as ethanol.

**Figure 3:**
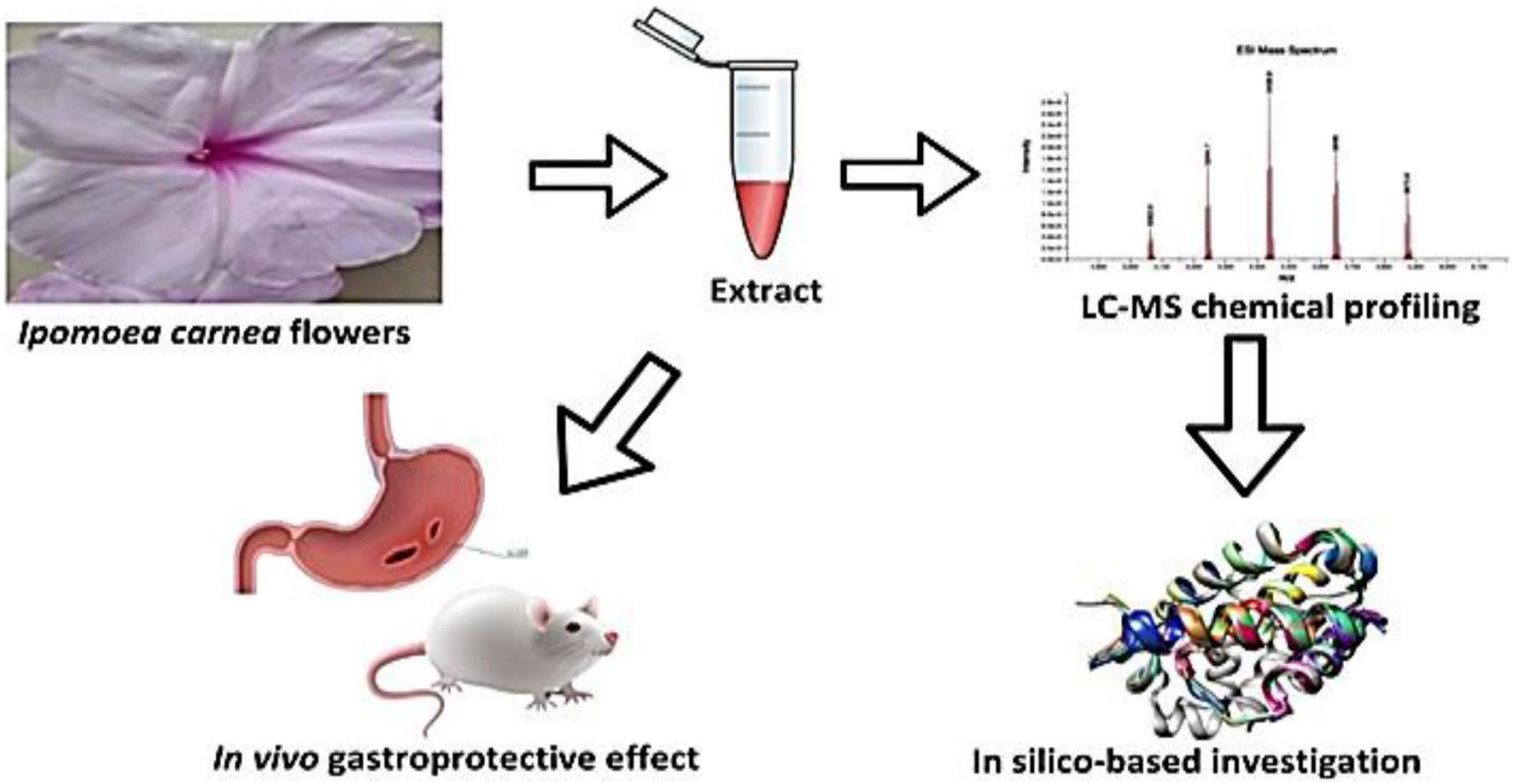
Graphical representation of the study.

By investigating the protective effects of a gastric protective extract in this specific model, the study aims to provide insights into the potential benefits of such interventions for individuals who face a dual challenge of radiation-induced gastric mucosal injury and subsequent ulceration. This innovative approach expands our understanding of the potential therapeutic strategies for preventing and treating gastric ulcers, particularly in individuals with a history of radiation exposure (Fig. 3).

## Results

### Quantitative phytochemical contents of ICME

Table 1 presented the results of the phenolic content analysis, indicating that the flower of *I. carnea* contained 76.43 ± 3.013 mg catechol equivalent per gram of dry extract. The presence of phenolics in the flowers has attracted significant attention due to their potential biological activities. Flavonoids, which represent a diverse and widely distributed group of natural compounds, are considered to be among the most important phenolic compounds. These compounds exhibit a wide range of chemical and biological activities. The analysis revealed that the flower of *I. carnea* contained 100.8 ± 3.68 mg quercetin equivalent per gram of dry sample in terms of flavonoid content. Additionally, the alkaloid content was determined to be 12.02 ± 0.299 mg per gram, while the tannin content was found to be 8.17 ± 1.20 mg tannic acid equivalent per gram.

**Table 1:** Total phenol, flavonoid, alkaloid, and tannin contents of ICME.

Test material	TPC mg CE/g D.W	TFC mg Qu/g D.W	TAC mg /g D.W	TTC mg TAE/g D.W
*I. carnea* flowers	76.43 ± 3.013	100.8 ± 3.68	12.02 ± 0.299	8.17 ± 1.20

Results were expressed as Mean ± SE of three replicates. TPC: Total phenolic content; TFC: Total flavonoid content; TAC: Total alkaloid content; TTC: Total tannin content. Qu: quercetin equivalent; CE: Catechin equivalent; TAE: Tannic acid equivalent. D.W.: Dry weight. mg/g D.W.): mg equivalent per gram of dry weight.

### Impact of Ipomoea carnea methanolic extract (ICME) on the stomach gross analysis

**Figure 4:**
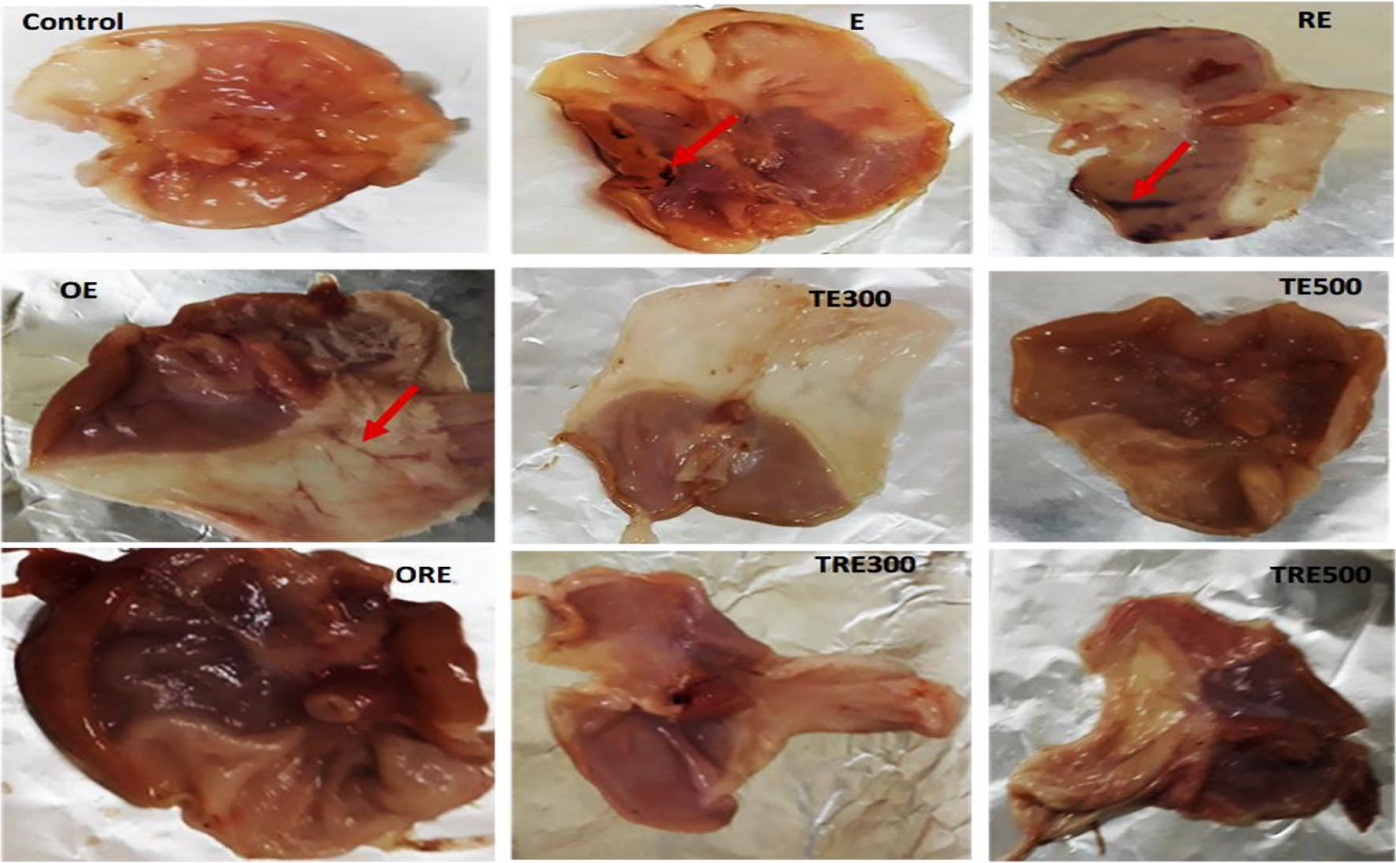
Effect of ICME on macroscopical appearance in ethanol-induced gastric mucosal injury in rats at necropsy. Normal group (Control), Ulcer group (E), Radiation group + Ulcer (RE), Omeprazole (OME; 20 mg/kg b.wt.) + Ulcer group (OE), ICME 300 mg/kg b.wt. + Ulcer group (TE300), ICME 500 mg/kg b.wt. + Ulcer group (TE500), OME + Radiation + Ulcer group (ORE), ICME 300 mg/kg b.wt. + Radiation + Ulcer group (TRE300), and ICME 500 mg/kg b.wt. + Radiation + Ulcer group (TRE500). A red arrow indicates a hemorrhagic lesion.

The pure ethanol (Fig. 4) caused severe damage to the stomach mucosa in both non-irradiated (E) and irradiated (RE) rats. The control group displayed no visible lesions. The GU group showed moderate ulcer damage compared to the healthy group, characterized by hyperemia in the glandular region, increased mucosal edema, and hemorrhage. The RE group exhibited more pronounced lesions in the gastric mucosa compared to the E group. However, rats treated with omeprazole (OME) and two different doses of ICME (300 and 500 mg/kg b.wt.) displayed mild lesions. These findings demonstrate that both non-irradiated and irradiated rats were significantly protected from ethanol-induced stomach ulcers by OME and ICME. The results indicate that the RE group exhibited a significant increase in stomach lesions in the submucosal linings compared to the other experimental groups. Conversely, the GU-irradiated rats (ORE, TRE300, and TRE500), pre-treated with ICME and OME, displayed significantly fewer ulcerated stomach lesions compared to the RE groups. Additionally, in both the E and RE groups, rats administered with ICME at a dose of 300 mg/kg b.wt. showed fewer mucosal injuries to the gastric epithelial surface compared to the ICME 500 mg/kg b.wt. group.

Overall, these findings highlight the gastroprotective effects of OME and ICME against ethanol-induced stomach ulceration in both non-irradiated and irradiated rats. The results suggest that ICME, particularly at a dose of 300 mg/kg b. wt., holds potential as a therapeutic agent for mitigating gastric mucosal injuries caused by ethanol and radiation exposure.

### Effect of ICME on the gastric pH

**Figure 5:**
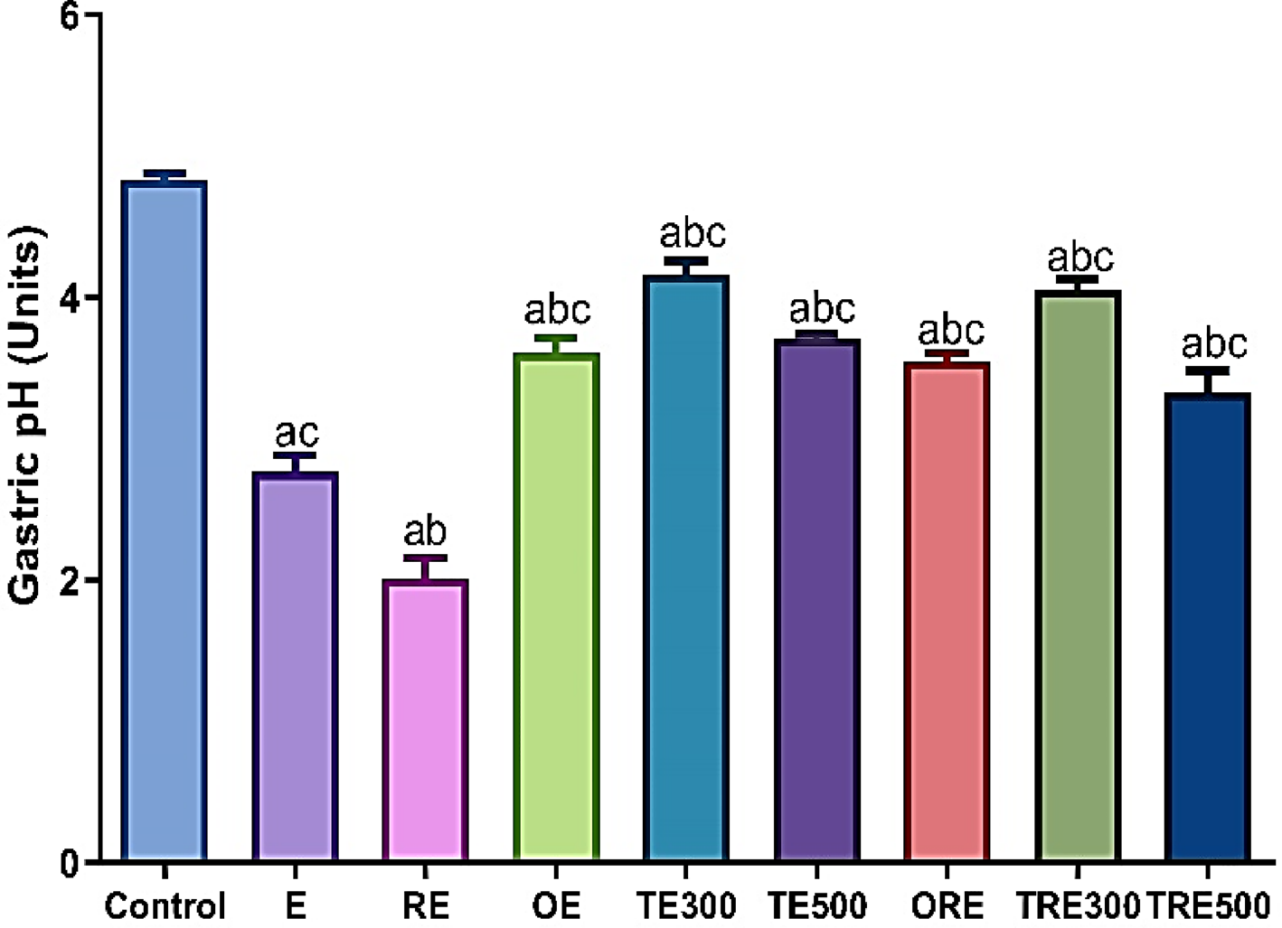
The effect of ICME on the gastric pH units of rats in ethanol-induced gastric ulcers for various groups. The data were presented as mean ± SEM (n = 6). (**a**) Significantly related to the control, (**b**) Significantly compared to the ethanol-induced gastric mucosal damages (E group), and (**c**) Significantly compared to the RE group by one-way ANOVA after Tukey’s post-hoc test (*p <*0.05).

Rats that were given ethanol-induced ulcers experienced a significant decrease in stomach pH compared to healthy rats (2.7 and 4.8, respectively). However, all pre-treated groups, whether with OME or ICME, showed a significant reduction in acidity compared to the group treated with ethanol (as shown in Fig. 5). Rats exposed to an acute radiation dose of 6 Gy before receiving ethanol exhibited a 25.5% reduction in stomach PH units (from 2.7 to 2.01 units) compared to the E group and a 58.12% reduction in gasstric PH units compared to control. Ethanol treated groups with ICME 300 and 500 mg/kg b.wt. (TE300 and TE500) showed a significant increase in gastric pH units by 34.14% and 27.02%, respectively, compared to the E group (*p <*0.0001). Additionally, both IR-pretreated groups with ICME 300 and 500 mg/kg b.wt. (TRE300 and TRE500) demonstrated a significant increase in gastric PH units compared to the RE group (50.37%; *p <*0.0001 & 39.45%; *p <*0.0001, respectively). Similarly, the groups of animals that received OME as the standard drug treatment before ethanol induction showed a significant increase in pH compared to the E ( from 2.7 to 3.6 units; *p <*0.0001) and RE groups (from 2.01 to 3.54 units; *p <*0.001). The ICME-treated group with a lower dose of 300 mg/kg b.wt. demonstrated significant restoration of pH levels nearly similar to the normal treatment group (*p <*0.0001).

### Effect of ICME on liver and kidney functions in normal and gastic ulcers induced rats

The current data demonstrated that the ethanolic groups (E and RE) had liver and kidney impairment and damage, as demonstrated by the significantly elevated serum hepatorenal biomarkers such as AST, ALT, urea, and creatinine with a reduction in total proteins. Rats treated with ICME (300 and 500 mg/kg b.wt.) and then ethanol in both irradiated and non-irradiated rats improved, and all altered parameters were restored (*p <*0.001), as shown in Table 2.

**Table 2:** Effect of ICME on the biochemical parameters of liver and renal function tests in experimental rats.

Groups	ALT	AST	Creatinine	Urea	Total protein
	(U/mL)	(U/mL)	(mg/dL)	(mg/dL)	(g/dL)
Control	61.03 ± 4.18	127 ± 3.0	0.54 ± 0.01	36.47 ± 0.33	0.13 ± 0.004
E	112.6 ± 3.54^ac^	177.8 ± 14.9	1.16 ± 0.009^ac^	72 ± 2.02^ac^	0.11 ± 0.006
RE	128.1 ± 6.36^a^	195.2 ± 9.8^a^	1.38 ± 0.01^a^	108.1 ± 5.01^ab^	0.11 ± 0.001
OE	91.66 ± 3.00^abc^	147.3 ± 8.1	0.67 ± 0.10^c^	61.41 ± 0.84^ac^	0.14 ± 0.004
TE300	80.16 ± 3.52^abc^	131.6 ± 14.8^c^	0.49 ± 0.07^bc^	35.15 ± 2.8^bc^	0.17 ± 0.005^abc^
TE500	85.8 ± 4.19^abc^	156.4 ± 6.8	0.57 ± 0.02^bc^	57.5 ± 6.2^ac^	0.12 ± 0.002
ORE	91.4 ± 2.02^abc^	159.8 ± 10.3	0.45 ± 0.05^bc^	60.29 ± 0.37^ac^	0.15 ± 0.004^bc^
TRE300	80.36 ± 3.93^abc^	151.2 ± 15.4	0.27 ± 0.01^abc^	48.7 ± 1.76^bc^	0.16 ± 0.006^bc^
TRE500	105.2 ± 0.90^ac^	172.1 ± 3.8	0.43 ± 0.04^bc^	65.4 ± 3.0^ac^	0.13 ± 0.01

The data were presented as mean ± SEM. Values are statistically significant when p ≤ 0.05. ^a^Significant compared to the control group ^b^Significant compared to the E group, ^c^Significant compared to the RE group (one-way ANOVA, post-hoc test, Tukey test). One international unit (U) is the amount of enzyme that transforms 1 µmol of substrate per minute. ALT, alanine aminotransferase; AST, aspartate aminotransferase.

### Biochemical investigation of stomach tissues in gastric ulcer conditions

**Figure 6:**
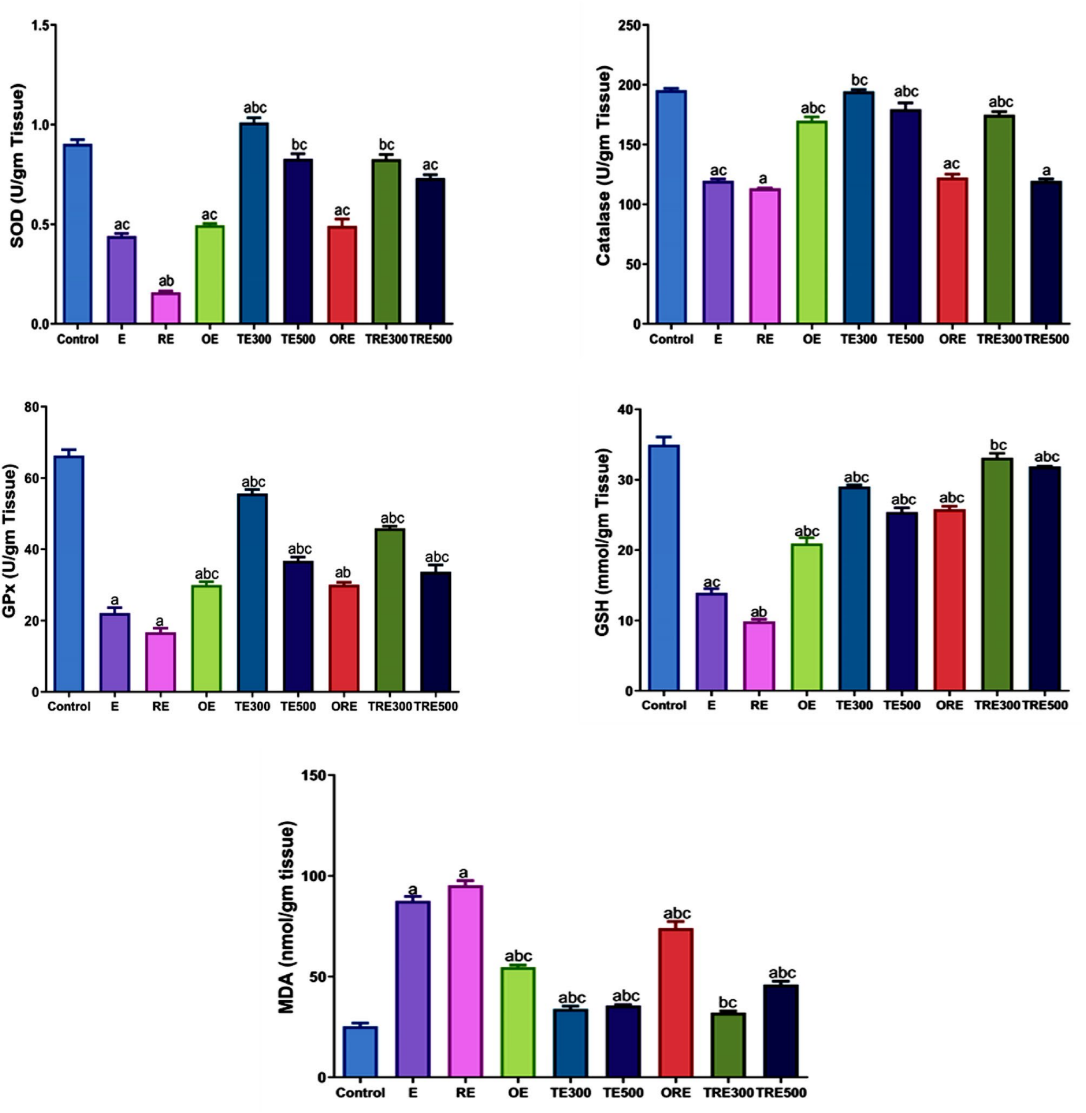
Effect of ICME on the gastric antioxidants of ethanol-induced gastric ulcers in non-irradiated and irradiated rats: superoxide dismutase (SOD) activity, catalase (CAT) activity, glutathione peroxidase activity (GPx), reduced glutathione level (GSH), and malondialdehyde (MDA) content. Values are expressed as the mean ± SEM (n = 6). (**a**) Significantly related to the control group. (**b**) Significantly compared to the E group, (**c**) Significantly compared to the RE group (one-way ANOVA, post-hoc test, Tukey test) at a p-value of 0.05.

The antioxidant levels (SOD, GSH, GPx, catalase, and MDA) in the stomach tissue homogenates were evaluated to examine the effect of antioxidant defenses on the gastric ulceration (GU) process. When compared to the control, ethanol-induced GU was linked to a considerable rise in MDA levels and a reduction in the values of the SOD, GPx, and catalase enzymes, as well as GSH concentration, in both irradiated and non-irradiated rats. OME (20 mg/kg b.wt.) and all doses of ICME (300 and 500 mg/kg b.wt.) significantly (*p <*0.001) improved the SOD, GPx, and catalase activities, increased GSH levels, and decreased MDA values when compared with the E and RE groups. ICME 300 mg/kg b.wt. dose achieved the best results in all parameter tests compared to ICME 500 mg/kg b.wt. dose and OME (Fig. 6)**.**

### Effect of ICME on Nrf2 and HO−1 levels in ethanol-induced gastric ulcers in both irradiated and non-irradiated rats

**Figure 7:**
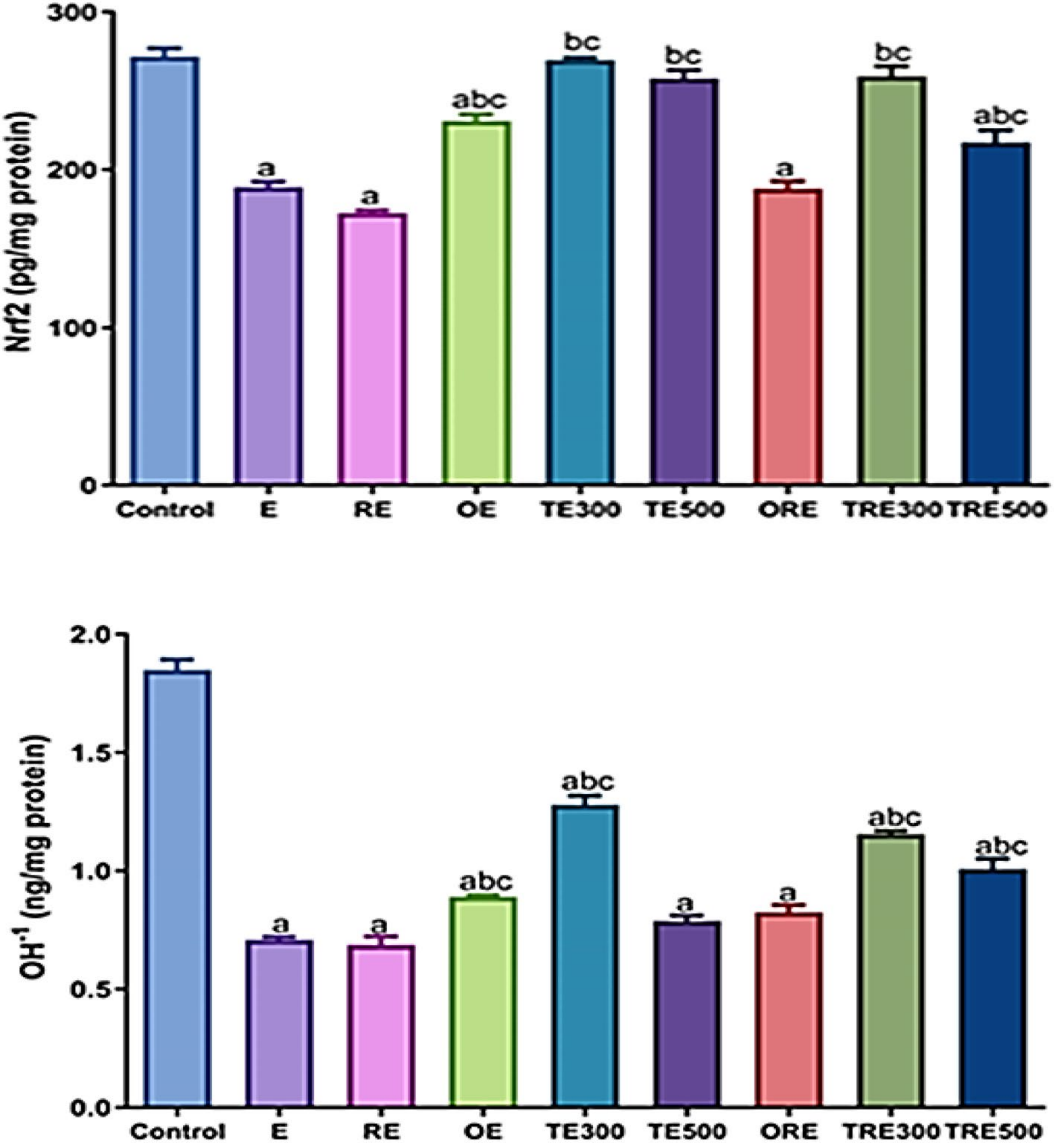
Effects of ICME (300 and 500 mg/kg b.wt.) treatments in acute gastric mucosal damage induced by ethanol in rats on Nrf2 and HO‐1 expression levels. All data were expressed as mean ± SEM (n = 6). (**a**) Significantly related to the control, (**b**) Significantly compared to the ethanol-induced gastric mucosal damages (E group), and (**c**) Significantly compared to the RE group by one-way ANOVA after Tukey’s post hoc test (*p <*0.05).

Rats were given ethanol displayed a significant decrease (*p <*0.001) in tissue levels of Nrf2 (30.42%; *p <*0.0001 and 36.6%; *p <*0.01) and HO−1 (61.9% and 63.04%; *p <*0.0001) in both non-irradiated (E group) and irradiated rats (RE group), respectively as in comparison to control rats. While, following ICME treatment at both doses (300 and 500 mg/kg b.wt.), rats showed upregulation of Nrf2 (29.85%; *p <*0.0001 and 26.64%; *p > *0.0001) and in HO−1 (45.26%; *p <*0.0001 and 10.25%; *p > *0.05) in gastric tissues when compared to ulcerated rats (E group). Moreover, results from rats treated with ICME (300 and 500 mg/kg b.wt.) were increased (33.41%; *p <*0.0001 and 20.57%; *p <*0.0001) for Nrf2 and (40.86%; *p <*0.0001 and 31.87%; *p <*0.0001) for HO-1, respectively related to the RE group. This demonstrated that the antioxidant defense system had been restored. Similar circumstance, treatment with OME as a reference drug revealed a similar increase in Nrf2 (18.13% and 8.24%; *p > *0.05) and HO−1 (21.3%; *p <*0.01 and 17.07%; *p > *0.05) when associated with the E and RE groups, respectively (Fig. 7)**.**

### Effect of ICME on body weight change and relative organ weight

**Figure 8:**
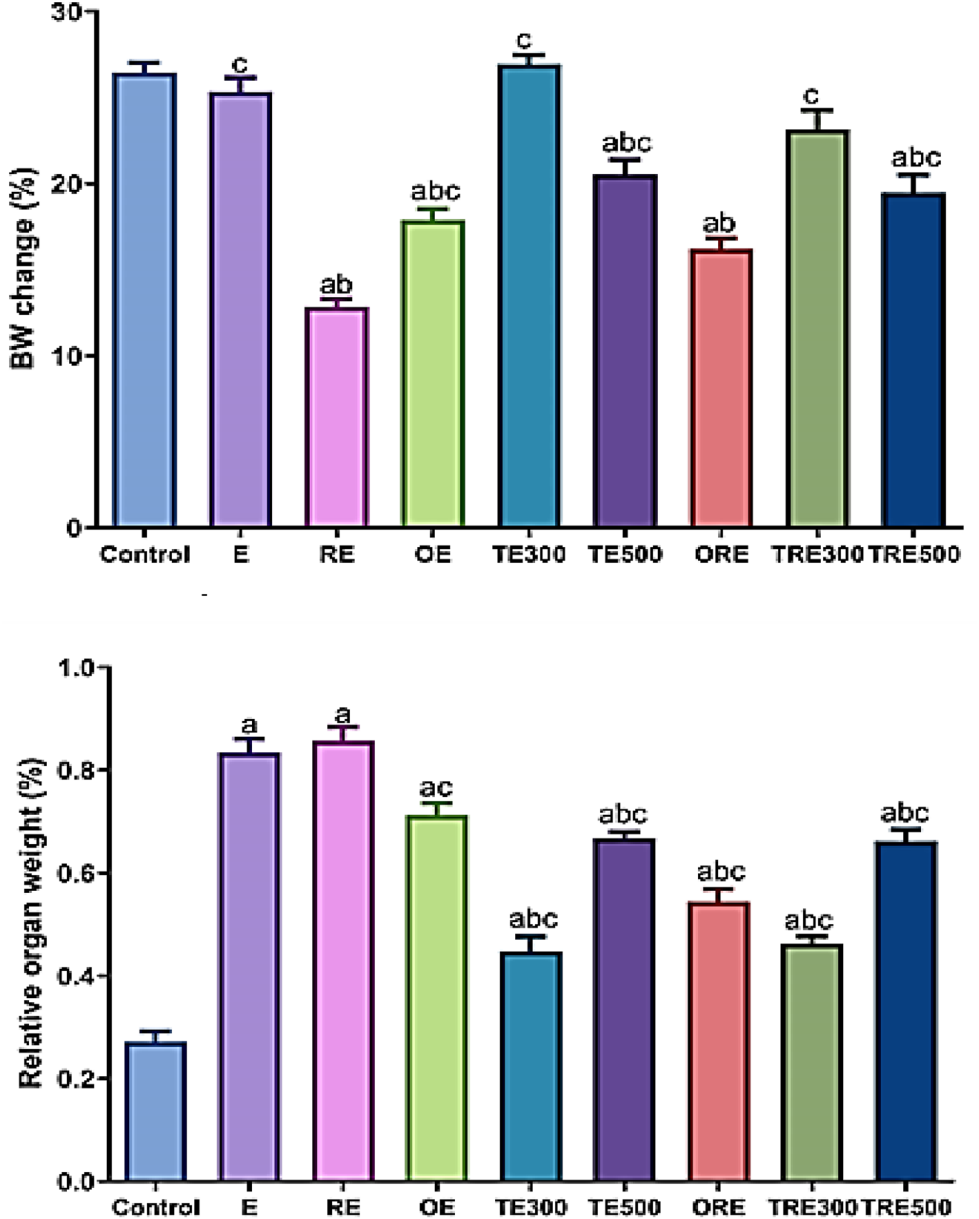
Effect of ICME on the relative stomach weight of the ulcer-induced rats. All values were presented as mean ± SEM at *p <*0.05.). (**a**) Significantly related to the control, (**b**) Significantly compared to the ethanol-induced gastric mucosal injuries (E group), and (**c**) Significantly compared to the RE group by one-way ANOVA after Tukey’s post hoc test (*p <*0.05).

The results on the impact of ICME on body weight change (%) and the relative stomach weight on ethanol induced GU of the irradiated and non-irradiated rats were demonstrated in Fig. 8**.** A non-significant difference in body weight percentage change (BW%) was found between the E and normal control groups (*p > *0.05). While there was a significant reduction in the BW (%) in the RE group related to the control. Treatment with ICME (TRE300 and TRE500) significantly increased BW% related to the RE group, *p <*0.001. On the other hand. The relative stomach weight of the ulcer-induced rats increased significantly more than that of the control group, *p <*0.0001. Nonetheless, a noticeable decrease in the relative weight of the stomach was observed in all rats administered with ICME (TE300 and TE500) as compared to the E-induced group.

### Effect of ICME on histopathological evaluation of the gastric mucosa in ethanol-treated, irradiated, and non-irradiated rats

**Figure 9:**
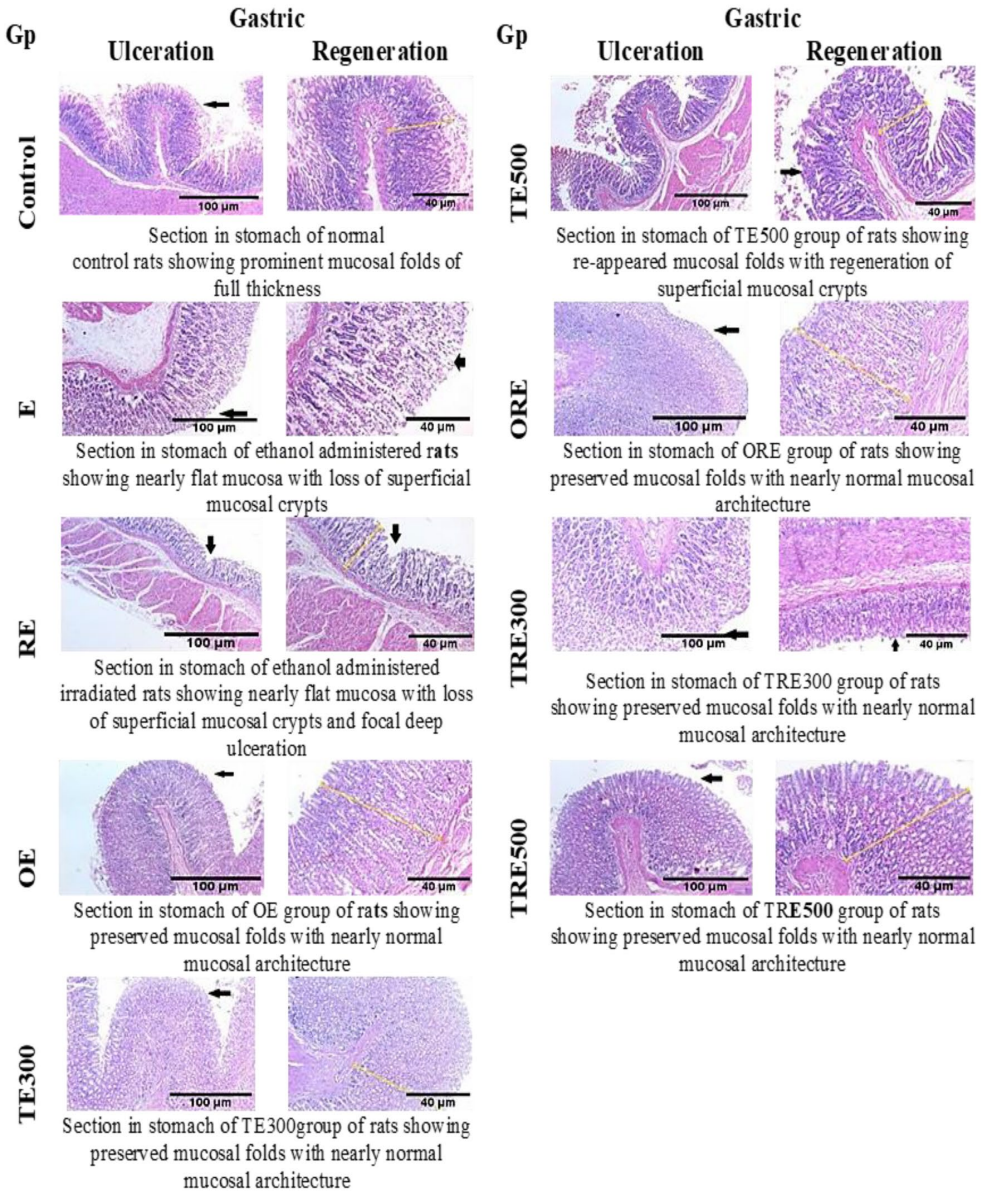
Influences of the ICME on the histological evaluation of the stomach tissues from rats subjected to ethanol-induced ulceration (H & E stain, magnification at ×100 on the left side and ×200 on the right side for all groups). According to microscopic images, the control showed normal mucosal anatomy. The ulcer groups (E and RE) had numerous lesions and extensive stomach mucosal damage; leukocytes and oedema were seen in the submucosal regions of the reference therapy (omeprazole, 20 mg/kg b.wt.), which caused mild mucosal damage; According to microscopic views, rats given 300 mg/kg b.wt. of the ICME exhibited mild mucosal damage but no oedema, while rats given 500 mg/kg b.wt. of the ICME had moderate mucosal lesions.

The histological analysis of the stomach mucosa is shown in Fig. 9**.** In the untreated group's stomach photomicrograph, the usual histological layers of the stomach mucosa are visible, which comprise glandular tissue, submucosa, muscular layer, and serosa covering. Moreover, ethanol-induced gastric ulceration (E group) was accompanied by mucosal damage, practically flat mucosa, and the loss of superficial mucosal crypts, as seen in a photomicrograph of the stomach. The photomicrograph of the stomach in the ethanol- and gamma-irradiated rats (RE group) revealed focal deep ulceration and practically flat mucosa, with loss of superficial mucosal crypts. An OE group of rats' stomachs were photomicrographed, and the mucosal architecture appeared to be nearly normal and had been retained. After the ethanol gavage, the rats that had not received any pre-treatment developed significant lesions with submucosal destruction (E and RE groups). Additionally, compared to the E group, the RE group displayed more significant subcutaneous edema with leukocytes. A greater defensive gastric epithelium, less stomach injury, and less inflammation and edema in the submucosa were observed in the ICME-treated rats. Additionally, the TE300 group's stomach portion revealed normal glands in the lamina and intact gastric mucosa without ulcers. The stomach sections of the ORE, TRE300, and TRE500 groups of rats showed preserved mucosal folds with nearly normal mucosal architecture, in contrast to the photomicrograph of the stomach of the TE500 group of rats, which demonstrated reappearing mucosal folds with the regeneration of superficial mucosal crypts. In the current study, analysis of the stomachs of control rats and all treated groups showed substantially identical histological structures of the gastric mucosa. Rats treated with OME and ICME at a low dose (300 mg/kg b.wt.) have greater preservation against stomach ulceration than rats treated with ICME at a higher dose (500 mg/kg b.wt.), as evidenced by decreased submucosal edoema, the depression of the ulcer area and the reduction of leucocyte infiltration. This showed that the pathological alterations in an ethanol-induced gastric ulcer were considerably reduced by ICME at 300 mg/kg b.wt..

### Effect of ICME on the histopathological changes in the kidney and liver tissues of rats (acute toxicity)

**Figure 10:**
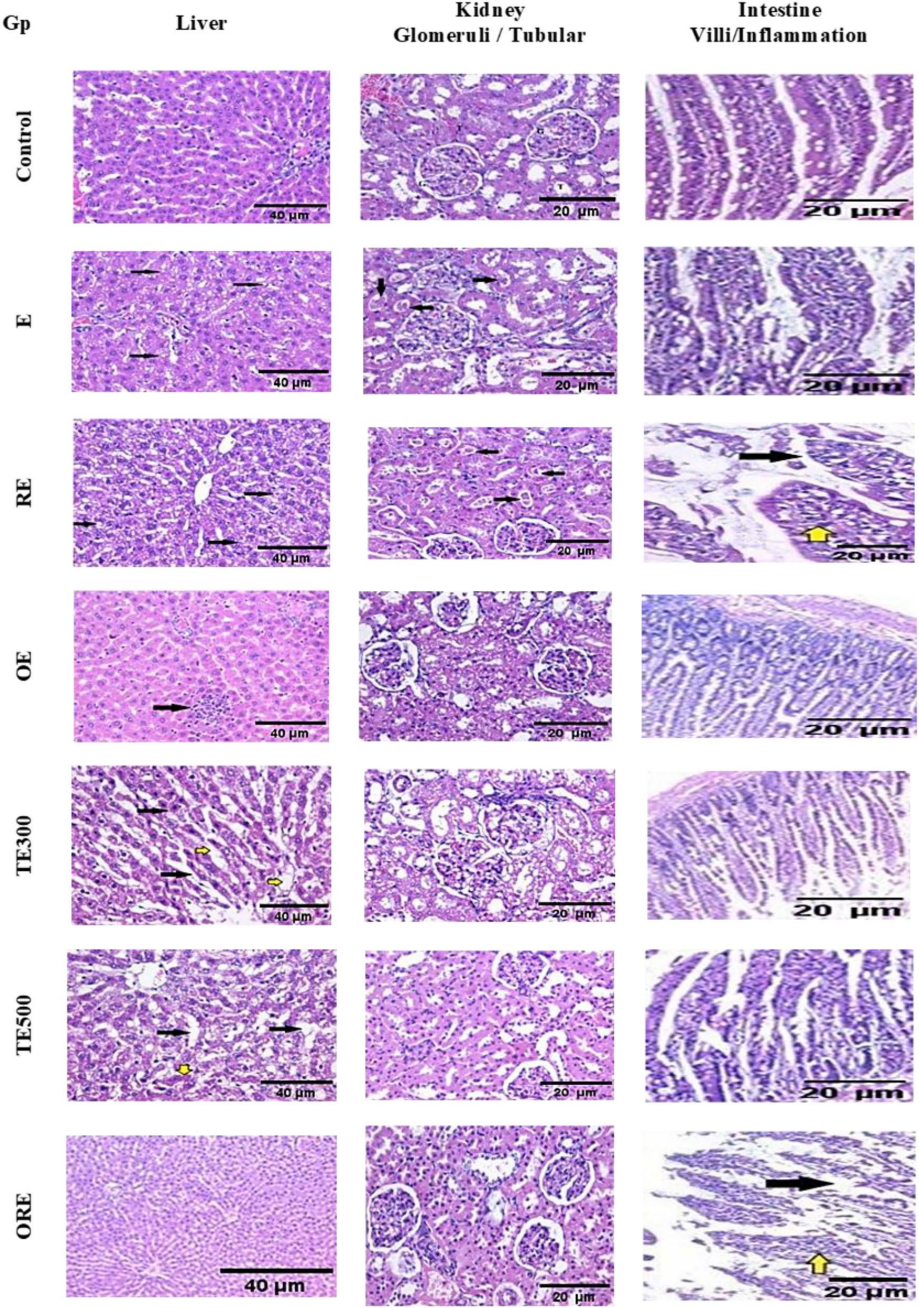
Histological examination of liver (H & E staining, magnification at ×200), kidney (H & E staining, magnification at ×400), and intestine (H & E staining, magnification at ×200) tested for acute toxicity in all experimental groups.

In the current study, examination of the livers of control rats revealed normal hepatic lobular architecture (Fig. 10). Sections of the liver of ethanol-administered rats (E) revealed pericentral intrahepatocyte microvesicular steatosis. On the other hand, liver sections of RE rats showed mild hepatocellular hydropic degeneration. Moreover, liver sections from group OE showed focal intralobular inflammatory cellular infiltration. A section in the liver of the TE500 group of rats demonstrated regeneration of hepatic cords with dilated sinusoids. However, liver sections from the TE300, ORE, TRE300, and TRE500 groups exhibited mild hydropic changes in hepatocytes. Furthermore, examination of the kidneys of control rats demonstrated normal cortical glomeruli and tubules. While the kidney of the E group showed an almost glomerular and tubular pattern, that of the RE group displayed unremarkable glomeruli with many intratubular hyaline casts. The microscopic kidney sections in the TE300, TE500, OE, ORE, and TRE300 groups revealed minimal histopathological changes at the light microscopic level. In addition, the TRE500 group showed focal cortical infiltration by mononuclear inflammatory cells. In addition, villous length, epithelial cells, and core pattern were also normal in intestinal sections from normal control rats. In the intestine of the E group, villous length was nearly normal with mild inflammatory cellular infiltration of the villous core. In contrast, sections from the intestine of the RE group revealed degenerated villous epithelium and moderate inflammatory cellular infiltration. Meanwhile, the OE group's intestines displayed nearly preserved villous architecture. The section in the intestine of the TE300 group showed mild degeneration of the villous epithelium. Also, the intestinal sections from the TE500 and ORE groups demonstrated marked degeneration of the villous epithelium. However, a section in the intestine of groups TRE300 and TRE500 demonstrated moderate degeneration of the villous epithelium.

### Chemical characterization of ICME

**Figure 11:**
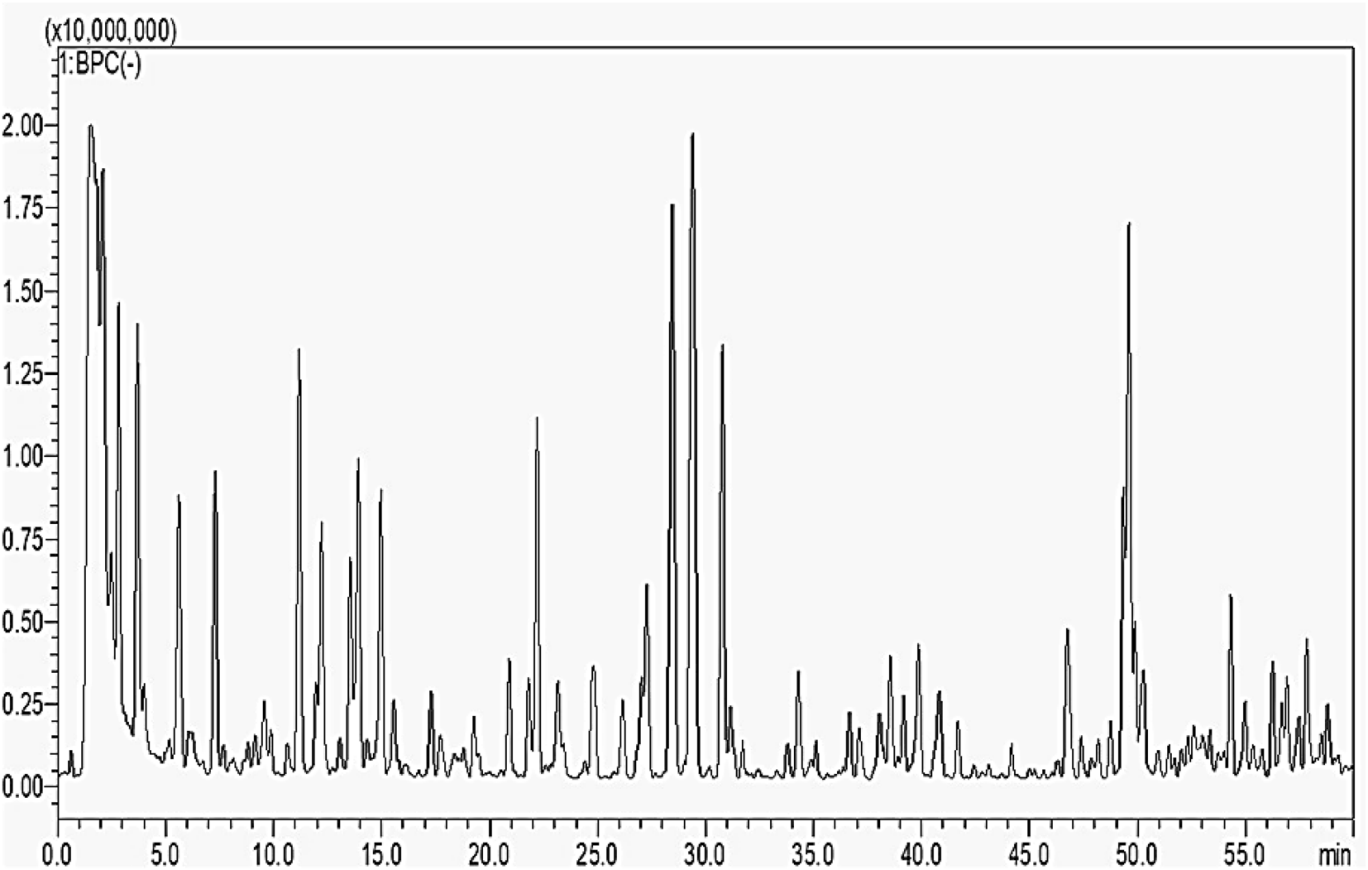
Negative ion BPC mass spectrum of *I. carnea* flower extract. (Peak numbers agree with those in Table 3).

The LCMS-8050 system with a triple quadrupole spectrometer and an ESI source is a powerful device that can measure both the quality and quantity of very small amounts of substances in complicated mixtures.Therity nine phenolics content in the *I. carnea* flower extract characterized by comparing their retention times and fragmentation patterns. Flavonoids and hydroxybenzoic acids were the two main phenolic subclasses detected in the investigated ICME. Organic and fatty acids are also included, among others (Fig. 11 and Table 3). Noticeably, a series of dihydroxybenzoic acid glycosides were detected, they provide an [M-H]^–^ *m/z* at 315, 299, 329, and 285 with identical daughter ions at 153 [M-H-162(Glu)]^–^, 153 [M-H-146(Rha)]^–^, 153 [M-H-176(Gluc)]^–^, and 153 [M-H-132(Pent)]^–^; these compounds were designated as dihydroxybenzoic acid glucoside, dihydroxybenzoic acid rhamnoside, dihydroxybenzoic acid glucuronide, and dihydroxybenzoic acid pentoside, respectively. Another characteristic set of quinic acid derivatives were also detected, they provided an [M-H]^–^ *m/z* at 337, 367, 515, 529, and 681 with daughter ions at 191[M-H-146 (coumaric moiety)]^–^, 193 [M-H-174 (quinic acid moiety)]^–^, 353 [M-H-162 (caffeic acid moiety)]^–^, 191 [M-H-338 (ferulic + caffeic acid moieties)]^–^, and 191[M-H-490 (ferulic + caffeic + gallic acid moieties)]^-^; these compounds were designated as coumaroylquinic acid, feruloylquinic acid, dicaffeoylquinic acid, feruloyl caffeoylquinic acid, and feruloyl galloyl caffeoylquinic acid, respectively. Additionally, a group of flavonoid compounds were detected; they provided an [M-H]^–^ *m/z* at 593, 625, 609, 447, and 593, with daughter ions at 305 [M-H-288 (catechin moiety)]^-^, 301 [M-H-324 (2 Glu)]^–^, 285 [M-H-324 (2 Glu)]^–^, 285 [M-H-162 (Glu)]^–^, and 285 [M-H-308 (Glu + coumaric moieties)]; these compounds were designated as (epi)gallocatechin-(epi)catechin, quercetin diglucoside, kaempferol diglucoside, kaempferol glucoside, and kaempferol coumaroyl glucoside, respectively.

**Table 3:** Phytoconstituents of *I. carnea* flower extract characterized by LC–ESI–MS/MS.

No	Rt	[M-H]^-^	MS/MS	Identified compounds	Chemical class	References
1	1.1	191	111	Citric acid	Organic acids	^38^
2	1.57	133	115	Malic acid	Organic acids	^39^
3	3.13	331	169	Galloyl glucose	Tannins	^40^
4	3.62	315	153	Dihydroxybenzoic acid glucoside	Phenolic acid derivatives	^41^
5	3.74	299	153	Dihydroxybenzoic acid rhamnoside	Phenolic acid derivatives	^41^
6	5.12	329	153	Dihydroxybenzoic acid glucuronide	Phenolic acid derivatives	^41^
7	6.20	153	109	Dihydroxybenzoic acid	Phenolic acids	^42^
8	7.04	341	179	Caffeoyl glucose	Phenolic acid derivatives	^43^
9	7.16	353	191	Chlorogenic acid	Phenolic acids	^44^
10	8.13	285	153	Dihydroxybenzoic acid pentoside	Phenolic acid derivatives	^45^
11	8.60	339	177	Aesculetin glucoside	Coumarins	^46^
12	11.06	353	191	Chlorogenic acid isomer	Phenolic acids	^47^
13	12.09	353	191	Chlorogenic acid isomer	Phenolic acids	^47^
14	12.27	355	193	Feruloyl glucose	Phenolic acid derivatives	^48^
15	13.53	593	305	(epi)Gallocatechin-(epi)catechin	Flavonoids	^49^
16	14.43	285	167	Phenyl acetyl vanillic acid	Phenolic acid derivatives	–
17	14.85	315	153	Dihydroxybenzoic acid glucoside	Phenolic acid derivatives	^41^
18	15.33	337	191	Coumaroylquinic acid	Phenolic acids	^39^
19	16.83	355	193	Feruloyl glucose	Phenolic acid derivatives	^48^
20	17.61	367	193	Feruloylquinic acid	Phenolic acid derivatives	^41^
21	18.56	163	119	Coumaric acid	Phenolic acids	^40^
22	19.18	625	301	Quercetin diglucoside	Flavonoids	^50^
23	20.70	609	285	Kaempferol diglucoside	Flavonoids	^50^
24	26.93	447	285	Kaempferol glucoside	Flavonoids	^51^
25	27.17	515	353	Dicaffeoylquinic acid	Phenolic acid derivatives	^52^
26	28.20	515	353	Dicaffeoylquinic acid	Phenolic acid derivatives	^52^
27	28.31	447	285	Kaempferol glucoside	Flavonoids	^51^
28	30.60	515	353	Dicaffeoylquinic acid	Phenolic acid derivatives	^52^
29	33.72	529	191	Feruloyl caffeoylquinic acid	Phenolic acid derivatives	^53^
30	36.49	529	191	Feruloyl caffeoylquinic acid	Phenolic acid derivatives	^53^
31	37.03	597	285	Kaempferol derivative	Flavonoids	-
32	38.12	681	191	Feruloyl galloyl caffeoylquinic acid	Phenolic acid derivatives	^53^
33	39.19	529	191	Feruloyl caffeoylquinic acid	Phenolic acid derivatives	^53^
34	40.51	593	285	Kaempferol coumaroyl glucoside	Flavonoids	^54^
35	49.48	327	183	Octadecadienoic acid derivative	Fatty acids	–
36	53.93	311	183	Octadecadienoic acid derivative	Fatty acids	–
37	53.99	343	177	Octadecadienoic acid derivative	Fatty acids	–
38	55.61	311	183	Octadecadienoic acid derivative	Fatty acids	–
39	55.66	293	177	Hydroxy-octadecatrienoic acid	Fatty acids	^55^

### In silico study

#### Virtual screening-based target identification

To determine how the *I. carnea* crude extract exerts its anti-inflammatory and ulcer protective activity, all of the LC–MS-annotated compounds' modelled structures were submitted to pharmacophore-based virtual screening utilizing the PharmMapper platform^56^. Pharm Mapper can track and suggest the most likely targets for proteins of the requested chemical depending on its pharmacophore description by mapping the main pharmacophore features (i.e., spatial arrangement of structural attributes).

As a result, compounds that adhere to these pharmacophore mappings are more likely to bind to the same protein targets. As a result, PharmMapper was used to identify possible protein targets for the identified chemicals in the *I. carnea* extract. The retrieved results were ranked based on how well they meet the requirements (i.e., Fit score). Only inflammatory-relevant and/or peptic ulcer relevant targets, were chosen.

**Figure 12:**
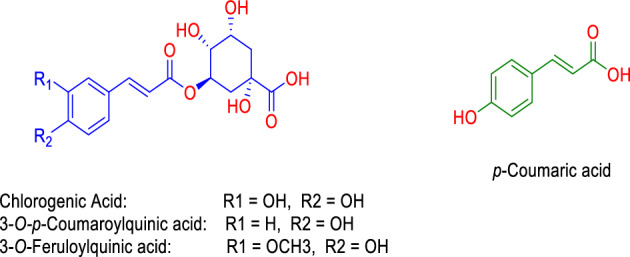
Structures that were found to be probably able to bind with NFκB (PDB ID: 1NFK; blue structures) and H^+^, K^+^-ATPase (PDB ID: 5YLV, green structure) according to the preliminary PharmMapper-based virtual screening alongside.

As a result, the nuclear factor kappa-B (NFκB; PDB ID: 1NFK) and the gastric proton pump (i.e., the H^+^, K^+^ -ATPase; PDB ID: 5YLV) were suggested to be the top-scoring inflammatory-relevant and ulcer-relevant hits for chlorogenic acid, 3-*O*-*p*-coumaroylquinic acid, 3-*O*-feruloylquinic acid, and *p*-coumaric acid, respectively (Fit scores = 11.82, 11.34, and 11.28, and 9.33 respectively; Fig. 12).

#### Molecular docking and dynamics simulation analysis

**Figure 13:**
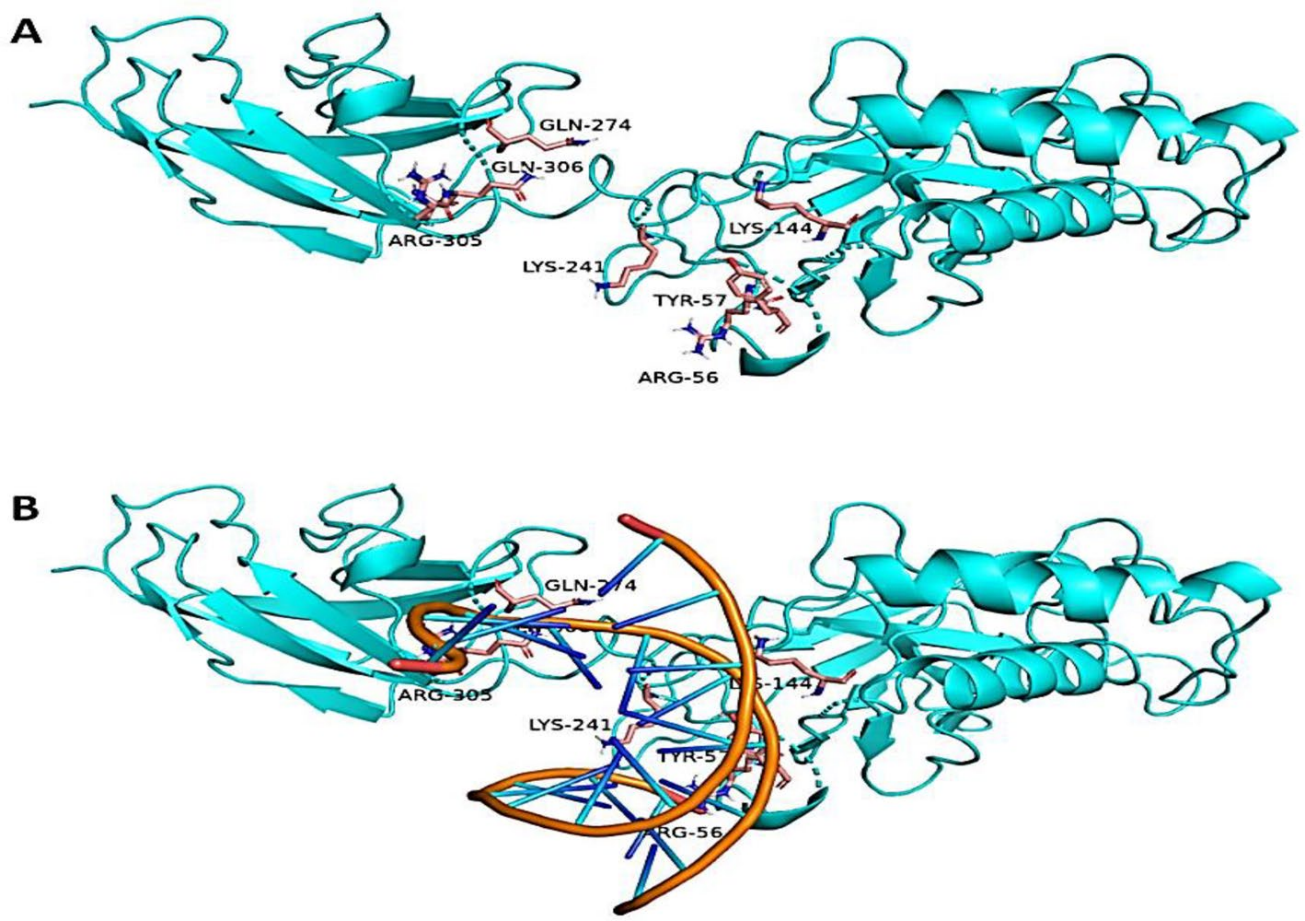
(**A**) Structure of NFκB (PDB ID: 1NFK) showing the key amino acid residues (showed in pink color) involved in DNA binding process and (**B**) how DNA interact inside this binding site.

To study the binding mechanisms of the aforementioned compounds (Fig. 13A) with NFκ-B and H^+^, K^+^-ATPase, their modelled structures were prepared and re-docked into each protein's active site. Following that, the retrieved binding poses were subjected to 100 ns-long MD simulation runs to validate each compound's binding stability inside the active sites of the indicated protein targets. NFκ-B is a transcription factor that binds with DNA, and hence its DNA-binding region (ARG-56, TYR-57, LYS-144, LYS-241, GLN-274, ARG-305, and GLN-306) was determined and used for the docking experiments (Fig. 13B).

**Figure 14:**
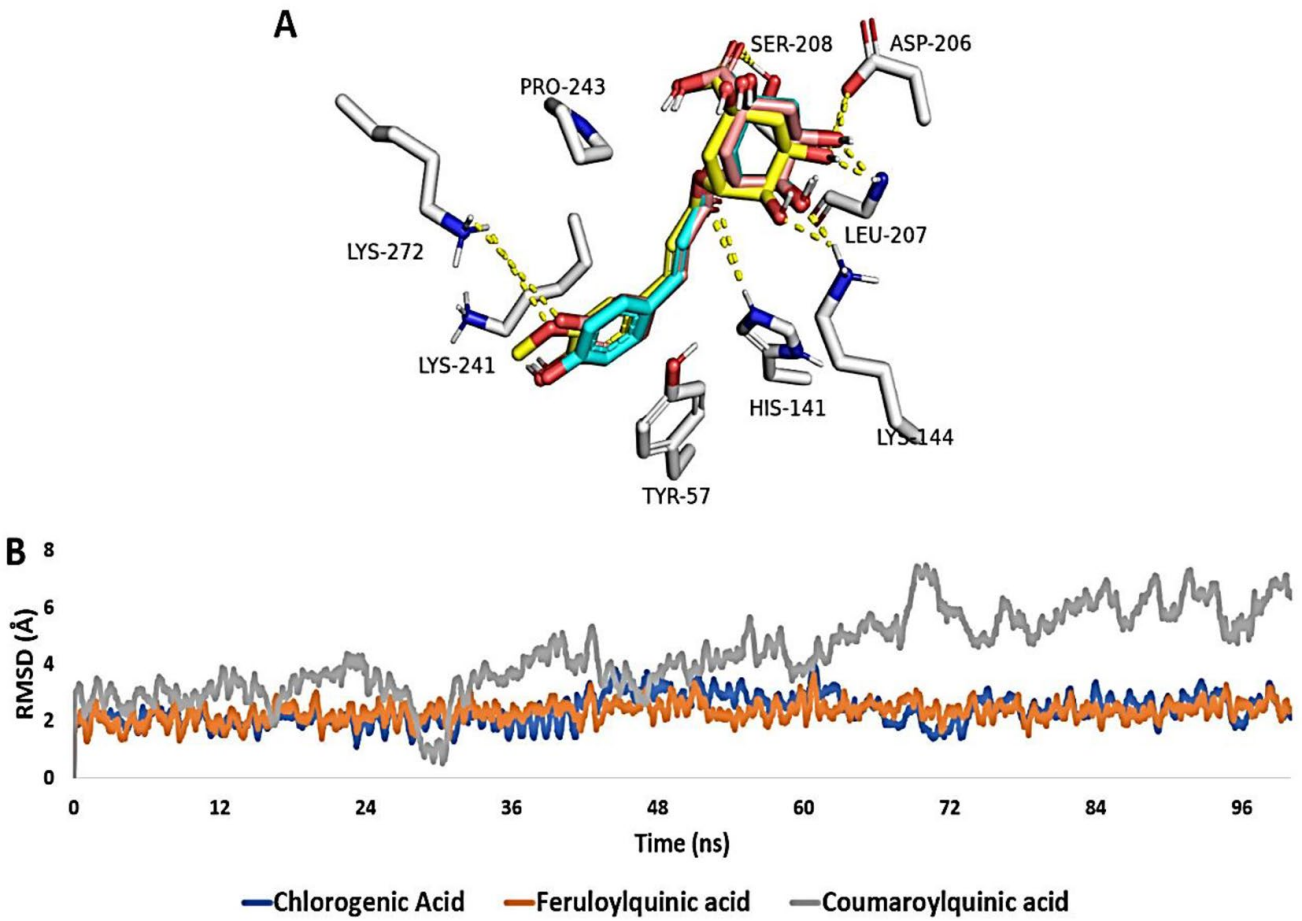
(**A**) Binding modes of chlorogenic acid (brick red-colored structure), 3-*O*-feruloylquinic acid (yellow-colored structure), and 3-*O*-*p*-coumaroylquinic acid (cyan-colored structure) inside NFκB’s active site. (**B**) RMSDs of aforementioned structures inside NFκB’s active site over the course of 100 ns-long MD simulation.

As shown in Fig. 14A and Table 4, chlorogenic acid, 3-*O*-feruloylquinic acid, and 3-*O*-*p*-coumaroylquinic acid were able to achieve almost identical binding modes inside NFκB’s active site. They also shared the same hydrophilic (i.e., H-bonding) and hydrophobic interactions except for 3-*O-p*-coumaroylquinic acid that did not for H-bonding with LYS-241due to the absence of the ortho-substituted hydroxyl groups. Accordingly, the calculated Δ*G*_Bind_ values were the same for both chlorogenic acid and 3-*O*-feruloylquinic acid, while it was significantly higher for 3-*O-p*-coumaroylquinic acid.

**Table 4:** Docking scores and ΔG_Bind_ (in kcal/mol) of chlorogenic acid, 3-*O*-feruloylquinic acid, and 3-*O*-*p*-coumaroylquinic acid inside NFκB’s active site, and *p*-Coumaric acid inside the H^+^, K^+^-ATPase’s active site. The table also shows the hydrophylic and hydrophobic interactions of each ligand inside the corresponding active site.

Tested compound	Docking score		MM-PBSA	H-Bonds			Hydrophobic interactions	
	NFκB	ATPase*	NFκB	ATPase*	NFκB	ATPase*	NFκB	ATPase*
Chlorogenic acid	− 8.27	–	− 7.21	–	HIS-141, LYS-144, ASP-206, ASP-207 SER-208, LYS-272	–	TYR-57, Lys-241	–
3-*O*-Feruloylquinic acid	− 8.15	–	− 7.18	–	HIS-141, LYS-144, ASP-206, ASP-207 SER-208, LYS-272	–	TYR-57, Lys-241	–
3-*O*-*p*-Coumaroylquinic acid	− 8.18	–	− 4.13	–	HIS-141, LYS-144, ASP-206, ASP-207 SER-208	–	TYR-57, Lys-241	–
*p*-Coumaric acid	–	− 10.46	–	− 10.01	–	ASN-138, GLU-795	–	LEU-141, VAL-338, TYR-799, ILE-816
Co-crystalized inhibitor**	–	− 12.28	–	− 10.13	–	LEU-811	–	LEU-141, VAL-338, TYR-799, ILE-816

* ATPase = H^+^, K^+^-ATPase; **The co-crystallized inhibitor for H^+^, K^+^-ATPase.

Moreover, both chlorogenic acid and 3-*O*-feruloylquinic acid modelled structures exhibited stable binding profiles over the course of 100 ns-long MD simulation (Fig. 14B) with an average RMSD of ~ 1.9 Å, while *3-O*-p-coumaroylquinic acid started to deviate significantly from its starting binding pose at ~ 47 ns to reach an RMSD value of 4.7 Å at the end of simulation. These results clearly indicate that the presence of ortho-substituted phenolic hydroxyl groups is very important for the stable binding of these scaffolds with NFκB. Additionally, the results proposed that both chlorogenic acid and *3-O*-feruloylquinic acid are likely NFκB inhibitors.

**Figure 15:**
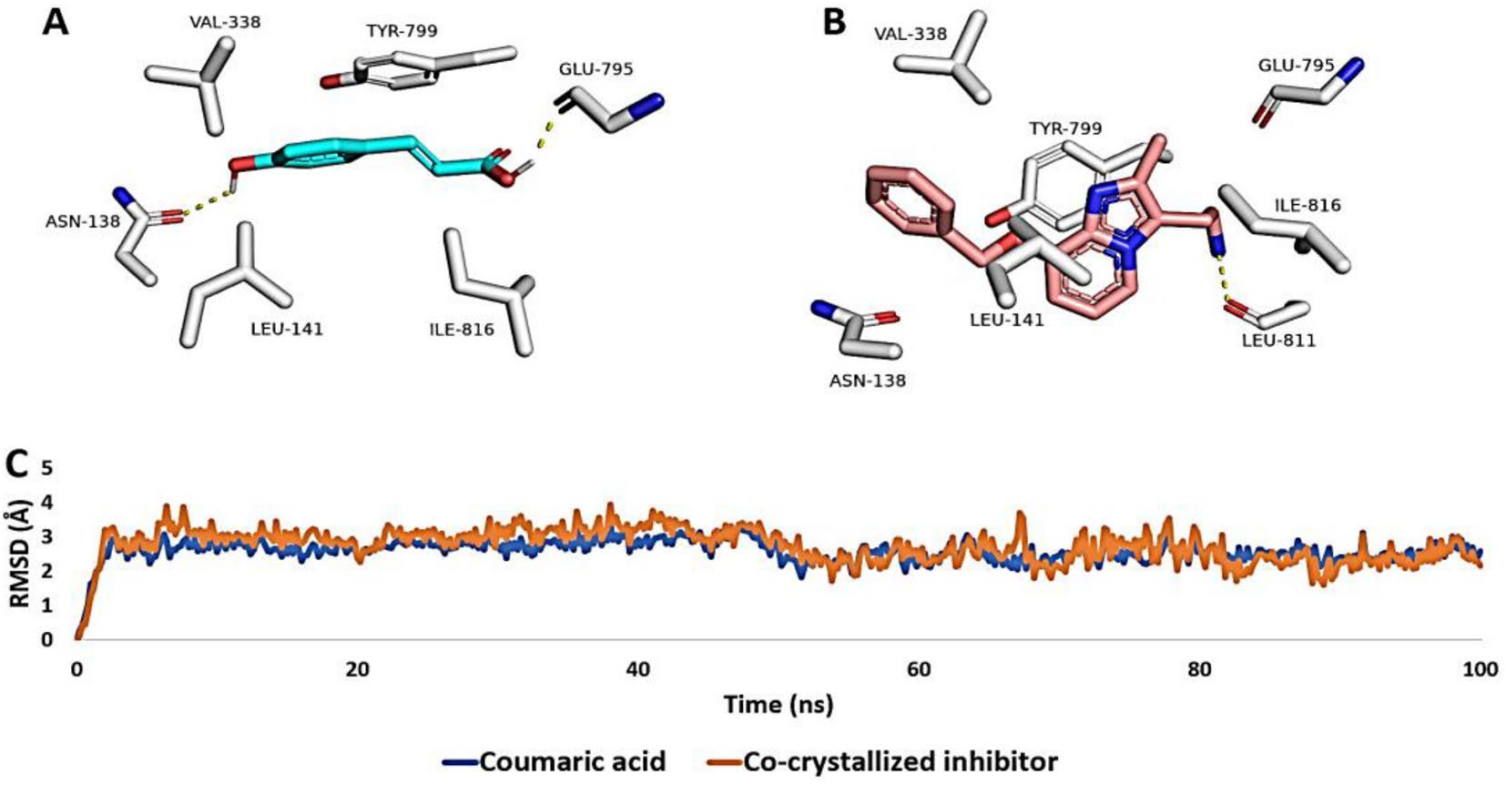
(**A, B**) Binding mode of *p*-coumaric acid (cyan-colored structure along with that of the co-crystallized inhibitor inside the H^+^, K^+^-ATP ase’s active site, respectively. (**C**) RMSDs of *p*-coumaric acid and the co-crystallized inhibitor inside the H^+^, K^+^-ATP ase’s active site over the course of 100 ns-long MD simulation.

On the other hand, *p*-Coumaric acid showed binding mode inside the H^+^, K^+^-ATPase’s active site that was comparable to that of the co-crystalized inhibitor (Fig. 15A,B, Table 4). Both structures shared the same hydrophobic interactions but differed in the hydrophilic ones (Table 3). Their calculated Δ*G*_Bind_ values were almost the same (Table 4), and hence their binding stability profile over the course of a 100 ns-long MD simulation was almost identical (Fig. 15C). Accordingly, *p*-coumaric acid can likely bind with and inhibit H^+^, K^+^-ATPase (Supplementary files).

Taken together, we can conclude that the glycosylated and non-glycosylated cinnamic acid derivatives annotated in *I. carnea* crude extract might be the key active principles that are responsible for the antiulcer efficacy of the extract. Additionally, these cinnamic acid derivatives might act via two independent mechanisms: (i) reducing the inflammatory response of the ulceration process, and hence, this can enhance the healing process; (ii) blocking the gastric proton pump making the gastric environment less hostile.

## Discussion

Gastric ulcers (GU) arise from an imbalance between the protective and destructive factors within the gastric system and current treatment options do not offer a definitive cure^57^. While several drugs are prescribed for peptic ulcer management, proton pump inhibitors are commonly used. However, their limitations, including incomplete curative effects, long-term adverse effects, and high cost, have prompted interest in investigating natural products as alternative treatments^58^.A powerful H + /K + ATPase inhibitor, omeprazole (the standard anti-ulcerative medication), has been widely used as a robust protection against many gastric ulcer models, including ethanol^59^. One of the main causes of GU include physiological stress and alcohol consumption. According to Abdallah et al.^60^, the pathophysiology of ethanol-induced GU in animals and human ulcers are identical. Ethanol exposure causes gastric lesions by entering and digesting the stomach wall as a result of its proteolytic and hydrolytic effect as well as by causing endothelial cell injury as a result of a decrease in blood circulation^61^.

In the present study, we established an experimental model of gastric ulcers induced by absolute ethanol at a dose of 5 mL/kg b.wt.. This model accurately represents various aspects of human gastric ulceration and is therefore valuable for evaluating the effectiveness of anti-ulcer drugs and identifying the underlying pathways these were in agreement with several previous studies^34,62^. Ethanol is one of the leading causes of gastric ulcers, and it inflicts damage through multiple pathways, including dehydration-induced disruption of mucosal cell barriers and cytotoxicity. The cytotoxic effects of ethanol contribute to the recruitment of leukocytes that release reactive oxygen species (ROS) and inflammatory cytokines, all of which may contribute to cell apoptosis^63^.

On the other hand, Ionizing radiation exposure can have significant impacts on stomach function in both rats and people. The stomach is a radiosensitive component of the gastrointestinal tract, making it unable to withstand the radiation doses required for cancer control. As it is frequently within the primary treatment field during radiotherapy, certain regions of the stomach can be affected by radiation, leading to the development of ulcers, perforation, chronic atrophic gastritis, and disruptions in secretory and motor functions^64^.

The present study aimed to investigate the gastroprotective effect of a phenolic-rich extract derived from *Ipomoea carnea* (*I. carnea)* flowers on ethanol-induced ulcers in irradiated and non-irradiated rats utilising omeprazole as a frequently prescribed medication. The study focused on elucidating the involvement of the Nrf2/HO−1 pathway in mediating the protective effects of the extract. Both in vivo and in silico evidence were employed to provide a comprehensive understanding of the underlying mechanisms.

The quantitative analysis of the phytochemical components in the flower extract of I. carnea has revealed significant quantities of phenolics and flavonoids, along with a smaller amount of alkaloids and tannins. These findings suggest that *I. carnea* contains a variety of bioactive compounds that may have potential therapeutic applications. This is consistent with the results of previous studies^65^.

Phenolic compounds and flavonoids derived from medicinal plants possess antiulcerogenic activity through various mechanisms, including anti-inflammatory, antioxidant, anti-H. pylori, and anti-secretory properties^66^. These compounds enhance prostaglandin synthesis, stress resistance, antioxidant enzyme production, and wound healing^67^. They also improve microcirculation, capillary resistance. Tannins directly directly protect the mucosa's top layer, making it more resistant to toxins and mechanical harm^68^. The presence of these active secondary metabolites in medicinal plants may contribute to a significant antiulcer and antioxidant effect. Oxidative stress, caused by an imbalance of free radicals and antioxidants, has been widely documented to cause and worsen peptic ulcers^69^. Additionally, oxygen-derived free radicals play a significant role in the pathogenesis of gastrointestinal mucosal damage^70^. The production of these reactive oxygen species is implicated in the enhanced lipid peroxidation in the gastric juice, which can lead to cell and tissue damag^71^.

Previous study showed that methanol exhibited the optimal solvent to extract the bioactive components since the highest content of phenolics, flavonoids, alkaloids, terpenoids, glycosides, saponin and amino acids were obtained^72^. The high polarity of methanol might have extracted vast number of polar phytochemicals from powdered *I. carnea* leaves and thereby increased the extract yield^73^. The methanolic extract of *I. carnea* had a higher TPC and GC–MS analysis revealed the presence of phytochemicals like 7‑methoxy coumarin, anethole, 8-prenylnaringenin and majdine which possess antioxidant, anti-inflammatory, anti-bacterial and angiogenic activities^15^.

Phenolic compounds have been recognized for their various health benefits, including their potential protective effects against gastric ulcers. Several studies have highlighted the gastroprotective and anti-ulcer properties of phenolic compounds. For instance, the methanolic extract of Melissa officinalis has been shown to have a gastroprotective effect against experimental gastric ulcers in rats, possibly due to its antioxidant activity^74^. Additionally, various polyphenolic compounds have been reported for their anti-ulcerogenic activity, acting as antisecretory, cytoprotective, and antioxidant agents^75^. These findings support the potential of phenolic compounds in the treatment of gastric ulcers. Isolating and identifying specific phenolic compounds from plant extracts and assessing their effects is necessary to fully comprehend their therapeutic potential and develop effective antiulcer drugs.

The exploration of *I. carnea* flower extract led to the characterization of phenolic compounds, which have anti-ulcer properties that depend mainly on the removal of free radicals and the suppression of oxidant enzymes^76^. Flavonoids have antispasmodic effects in the digestive tract, according to reports^77^, antiulcer, and antioxidant properties^78^. Kaempferol and quercetin-type glycosides showed antihistaminic characteristics, which lower histamine levels. This protective effect could be achieved through several methods. Quercetin is one of the most studied flavonoids; it protects the gastrointestinal mucosa from acute lesions induced by various experimental models and against different necrotic agents, including ethanol-induced gastric ulcers^79^. Quercetin works through an anti-secretory mechanism. It also inhibits the gastric H + /K + proton pump and prevents histamine from being released by gastric mast cells, which reduces the amount of acid secreted from the stomach^80^.

Cinnamic acid derivatives, such as chlorogenic acid and caffeoylquinic acid, have been studied for their biological efficacy and potential therapeutic applications. Previous works have evaluated plant extracts rich in cinnamic acid derivatives and have reported their beneficial effects, including antioxidant, anti-inflammatory, antimicrobial, anticancer, neuroprotective, and antidiabetic properties^81^. Previous study by Santos et al. reported the gastroprotective mechanism of cinnamic acid derivatives, such as chlorogenic acid and caffeoylquinic acid, against ethanol-induced gastric mucosal damage is multifactorial and possibly involves an antioxidant effect, stimulation of endogenous prostaglandins and nitric oxide release, activation of capsaicin-sensitive gastric efferents and K + ATP channels, accompanied by an increase in gastric mucus^82^.

Furthermore, the relative stomach weight can serve as an indicator of the overall health and condition of the stomach. Changes in stomach weight may reflect alterations in gastric motility, inflammation, or tissue damage, which are relevant factors in the context of ethanol-induced ulceration^83^. Our findings demonstrated extensive ulceration in rats after ethanol administration in both normal and irradiated rats in the form of a significant reduction in gastric PH, increases in stomach weight. In the present study, rats exposed to ethanol exhibited increased relative stomach weights, which were subsequently reduced by pretreatment with ICME at both dosage levels. As mentioned earlier^84^, acute ethanol exposure induces hemorrhage, edema, necrosis, and inflammation, all of which contribute to an increase in stomach weight. Additionally, no noticeable change in body weight was observed, which could be attributed to the sudden intake of ethanol.

In the present study, it was observed that the gastric juice pH in the ethanol-exposed stomach was significantly lower compared to the control stomach. However, pre-administration of ICME at both dosage levels and omeprazole at a dose of 20 mg/kg b.wt. significantly increased the pH, in contrast to the ulcer groups (E and RE). This finding is consistent with previous studies, which have documented that acute ethanol exposure leads to increased acid production, resulting in a decrease in the pH of gastric juice^85^.

additionally, the results of the current investigation showed that radiation exposure (6 Gy) impacts gastric acid secretion. Gastrin stimulates the production of acid, hence the marked gastrinemia brought on by irradiation may have contributed to the elevation in gastric acid secretion seen in the present study. According to Lehy et al. (1998), irradiation in rats increased basal (non-stimulated) stomach acid secretion along with an increase in plasma gastrin^86^. After radiation exposure, rats may experience acute erosive and ulcerative gastritis as well as atonic dilatation of the stomach^6^. However, Lehy et al. (1998) reported that rats exposed to 6-Gy whole-body gamma radiation had higher plasma levels of gastrin and increased stomach acid secretion^87^.

Moreover, in this study, it was observed that compared to the control group, E and RE groups showed significantly increase in the levels of malondialdehyde (MDA) while decreasing in the levels of antioxidants in the stomach were observed. However, when compared to the GU groups, pre-administration of ICME and omeprazole significantly reduced the MDA level and increased the levels of catalase (CAT), superoxide dismutase (SOD), glutathione peroxidase (GPx), and reduced glutathione (GSH). According to Liu et al., administering 100% ethanol results in the production of ROS, which further encourages lipid peroxidation in gastric tissue and results in severe damage to the gastric mucosal layers^88^. Also, the generation of ROS produces oxidative stress disturbances, which are manifested by a rise in the amount of TBARS and nitrite associated with a decrease in GSH. There is a strong relationship between oxidative stress and ethanol-induced gastric ulcers. Subsequently, reactive oxygen species react with lipids to generate lipid peroxides, producing large injury^89^.

ROS are frequently captured by the body's built-in antioxidant defense system, which also contains antioxidant enzymes like SOD and CAT. The transcription factor nuclear factor-erythroid-related factor 2 (Nrf2) strongly contributes to the mechanisms underlying cell abnormalities by maintaining the antioxidant capacity of cells^90^ and inhibits pro-inflammatory signalling by reducing NFκB^91^, The antioxidant enzymes heme oxygenase-1 (HO−1), CAT, and GSH are the subsequent features of Nrf2 signaling. According to a study, the Nrf2/HO−1 pathway enables HO−1 to maintain its protective properties against ethanol-induced ulcers^92^. In a previous work by Chou et al. (2013), it was found that pretreatment with *I. carnea* reduced ethanol-induced inflammatory responses by increasing HO−1 activity^93^. In the current investigation, pretreatment with *I. carnea* produced additional up-regulation of the levels of Nrf2 and HO−1. Management of therapeutic doses of *I. carnea* (300 and 500 mg/kg b.wt.) was studied to reveal which dose would be most ethanol-induced and efficient in the treatment of stomach ulcers. The study revealed that 300 mg/kg b.wt. of *I. carnea* was most effective in protecting against ethanol-induced gastric ulcers.

According to macroscopic and pathologic examinations of ulcer lesions, animals treated with ICME and OME had less stomach mucosa damage in ethanol. The histological examination in the present study revealed that the ulcer control group exhibited marked hemorrhagic injury to the gastrointestinal layers of the stomach, characterized by elevated neutrophil infiltration, penetration, and emphysema. These results were in agreement with previous studies which reported that ethanol administration causes histological changes in the stomach, including loss of gastric mucosa, epithelium integrity, vascular congestion, submucosal oedema, necrosis, and inflammatory response due to neutrophil and eosinophil infiltration^94^. When compared to the stomach mucosa, ICME in pretreated rats before ethanol ingestion caused less damage to the stomach, including fewer lesions and no edema. As aforementioned and according to our research, *I. carnea* extract pretreatment considerably enhanced the histopathological findings in a dose-dependent way, which is like the results following omeprazole administration (20 mg/kg b.wt.). This suggests that *I. carnea* may be helpful in the treatment of stomach ulceration.

It is worth noting that NFκ-B is involved in a variety of immunological and inflammatory actions. TNF-, IL-1, IL-6, IL-12, and cyclooxygenase-2 are all induced by NFκ-B p65, which is a crucial transcription factor of M1 macrophages^95^. MAPK/NFκ-B signal pathway-related proteins p-ERK, p-JNK, p-p38, p-IB, and p-NF-B p65 are thought to have protective effects in preventing gastric ulcer development^96^. Previous research has linked NFκ-B signalling to the development and development of gastric ulcer^97^. On the other hand, gastric proton pump is the key regulator of gastric acidity that can worsen gastric ulcers and slowdown the healing process^98^. Moreover, a number of cinnamic acid derivatives including p-Coumaric acid have been demonstrated potent in vitro H + , K + -ATPase activity^99^ indicating the validity of our previous in silico-based investigation.

## Conclusions

The present study annotated 39 compounds from the *I. carnea* flower extract using LC–ESI–MS/MS analysis. The findings of this study provide compelling evidence for the gastroprotective effect of the phenolic-rich extract from *Ipomoea carnea* (*I. carnea)* flowers on ethanol-induced ulcers in irradiated rats. The extract demonstrated its potential to mitigate oxidative stress, restore antioxidant defenses, and suppress inflammation through the activation of the Nrf2/HO−1 pathway. The in silico evidence further supported these observations by elucidating the molecular interactions between the extract's bioactive compounds and key targets involved in gastroprotection. These findings contribute to the growing body of knowledge on natural interventions for gastric ulcers and pave the way for future studies and the development of effective therapeutic strategies.

Bio-guided fractionation and isolation of the extract are strongly recommended as future perspectives. Further virtual screening-based and MD simulation-based investigations revealed that the cinnamic acid derivatives present in the extract of *I. carnea* might protect against gastric ulceration via reducing the generation of gastric acid (i.e., inhibiting H^+^, K^+^-ATPase) and the inflammatory mediators (i.e., NFκB).

## Supplementary Information

Supplementary Information.

## Data Availability

The authors confirm that the data supporting the findings of this study is available within the article. Raw data is accessible from the corresponding author on reasonable request.

## Abbreviations

ICME: *Ipomoea carnea* Methanolic extract
LC–ESI–MS/MS: Liquid chromatography electrospray ionization mass spectrometry
GU: Gastric ulcer
TEC: Total extractable content
HPLC–PDA-MS/MS: High-performance liquid chromatography-photodiode array detection-mass spectrometry
*m/z*: Mass-to-charge
Rt: Retention time
GPT: Alanine aminotransferase
GOT: Aspartate amino transaminase
CAT: Catalase
SOD: Superoxide dismutase
GSH: Glutathione reduced
GPx: Glutathione peroxidase
MDA: Malondialdehyde
Nrf2: Nuclear factor erythroid 2-related protein
HO-1: Hemoxygenase-1
NCRRT: National centre for radiation research and technology
OECD: Organization for economic cooperation and development's
LD_50_: Median lethal dose
OME: Omeprazole
AST: Serum aspartate transaminase
ALT: Alanine transaminase
ELISA: Enzyme-linked immunosorbent assay
ANOVA: Analysis of variance
