# Supplementary material for: Ipomoea carnea mitigates ethanol-induced ulcers in irradiated rats via Nrf2/HO−1 pathway: an in vivo and in silico study
Source: Sci Rep. 2024 Feb 12;14:3469. doi: 10.1038/s41598-024-53336-1 (PMC10859386; doi:10.1038/s41598-024-53336-1)
Supplement: Supplementary file 1 — Supplementary Information. [file 41598_2024_53336_MOESM1_ESM.docx]

**Supplementary Materials**

***Gastro-protective effect of phenolic-rich*** ***Ipomoea carnea flower extract*** ***on ethanol-induced ulcer in irradiated rats via Nrf2/HO-1 pathway: In vivo and in silico evidence***

**Mosad A. Ghareeb ^1,*^, Hala Sh. Mohammed ^2^, Tarek Aboushousha ^3^, Dina M. Lotfy ^4^, Maha A.M. El-Shazly ^1^, Mansour Sobeh ^5^ and Eman F.S. Taha^6^**

^1^Medicinal Chemistry Department, Theodor Bilharz Research Institute, Kornaish El-Nile, Warrak El-Hadar, Imbaba, P.O. Box 30, Giza 12411, Egypt; m.ghareeb@tbri.gov.eg (M.A.G.); m.elshazly@tbri.gov.eg (M.A.M.E)

^2^Department of Pharmacognosy and Medicinal Plants, Faculty of Pharmacy (Girls), Al-Azhar University, Cairo 11311, Egypt; Halashaaban676.el@azhar.edu.eg (H.S.M.)

^3^Department of Pathology, Theodor Bilharz Research Institute, Kornaish El-Nile, Warrak El-Hadar, Imbaba, P.O. Box 30, Giza 12411, Egypt; T.aboushousha@tbri.gov.eg (T.A.)

^4^Drug Radiation Research Department, National centre for Radiation Research and Technology, (NCRRT), Egyptian Atomic Energy Authority (EAEA), Cairo, Egypt; pharmadinaa@yahoo.com (D.M.L.)

^5^AgroBioSciences, Mohammed VI Polytechnic University, Lot 660, Hay Moulay Rachid, Ben-Guerir 43150, Morocco; mansour.sobeh@um6p.ma (M.S.)

^6^Health Radiation Research Department, National Centre for Radiation Research and Technology, (NCRRT), Egyptian Atomic Energy Authority, Cairo, Egypt; Eman.Fayez@eaea.org.eg (E.F.S.T.)

***Corresponding author: Eman FS Taha**

**E-mail**:  [emanfayezsaid@gmail.com](mailto:emanfayezsaid@gmail.com)

[Eman.Fayez@eaea.org.eg](mailto:Eman.Fayez@eaea.org.eg)

**1. *In silico* study**

**1.1. Virtual Target identification**

The putative targets characterization of the annotated compounds in the crude extract was achieved via Pharmacophore-based Virtual screening using PharmMapper [1]. This platform assigns a score to each molecule in the PDB that best fits a pharmacophore model that has been extracted and stored as a library of ligand dataset in mol2 format. After that, when a new molecule is submitted, its fit score is calculated for each pharmacophore, and then each fit score for that pharmacophore is compared to the fit score matrix to determine where it falls on the scale of all the pharmacophore scores. In comparison to chance pharmacophore matching, the pure fit score that results from this procedure carries considerably more weight and assurance. The query structure was submitted to the platform in the PDB format, and the retrieved results were exported as Excel sheet arranging the resulted protein targets according their fit scores.

***1.2. Docking Study***

The crystal structures of NFκB; PDB ID: 1NFK [2], and the H^+^, K^+^ -ATPase; PDB ID: 5YLV [3], were used for the docking study using AutoDock Vina [4]. The co-crystallized ligand in each structure was used to determine the binding site and the docking grid-box in each protein structure. The co-ordinates of the grid-box were set to be: x= -13.736, y= 13.816, z= 21.621; and x= 24.546, y= -16.356, z= 7.665, respectively. The ligand to binding site shape matching root means square (RMSD) threshold was set to 2.0 Å. The interaction energies were determined using the Charmm force field (v.1.02) with 10.0 Å as a non-bonded cut-off distance and distance-dependent dielectric. Then, 5.0 Å was set as an energy grid extending from the binding site.^4^ The tested compound retinol was energy minimized inside the selected binding pocket. The editing and visualization of the generated binding poses were performed using Pymol software [5].

**1.3. Molecular Dynamics Simulation**

NAMD 3.0.0. software was used for performing MDS [6,7]. This software applies the Charmm-36 force field. Protein systems were built using the QwikMD toolkit of the VMD software [7,8], where the protein structures were checked for any missing hydrogens, the protonation states of the amino acid residues were set (pH = 7.4), and the co-crystalized water molecules were removed. Thereafter, the whole structures were embedded in an orthorhombic box of TIP3P water together with 0.15 M Na^+^ and Cl^-^ ions in 20 Å solvent buffer. Afterward, the prepared systems were energy minimized and equilibrated for 5 ns. The parameters and topologies of the ligands were calculated by using the VMD plugin Force Field Toolkit (ffTK). Afterward, the generated parameters and topology files were loaded to VMD to readily read the protein–ligand complexes without errors and then conduct the simulation steps.

**1.4. Binding Free Energy Calculations**

Molecular Mechanics Poisson-Boltzmann Surface Area (MM-PBSA) embedded in the MMPBSA.py module of AMBER18 was utilized to calculate the binding free energy of the docked complex [9]. 100 frames were processed from the trajectories in total, and the system's net energy was estimated using the following equation:

ΔG_Binding_ = ΔG_Complex_ – ΔG_Receptor_ – ΔG_Inhibitor_

Each of the aforementioned terms requires the calculation of multiple energy components, including van der Waals energy, electrostatic energy, internal energy from molecular mechanics, and polar contribution to solvation energy.

**References**

1. Wang, X.; Shen, Y.; Wang, S.; Li, S.; Zhang, W.; Liu, X.; Lai, L.; Pei, J.; Li, H. PharmMapper 2017 update: A web server for potential drug target identification with a comprehensive target pharmacophore database. Nucleic Acids Res. 2017, 45, W356-W360. https://doi.org/10.1093/nar/gkx374.

2. Ghosh, G.; Duyne, G.V.; Ghosh, S.; Sigler, P.B. Structure of NF-κB p50 homodimer bound to a κB site. *Nature*.  **1995**, *373*, 303-310.‏ <https://doi.org/10.1038/373303a0>.

3. Abe, K.; Irie, K.; Nakanishi, H.; Suzuki, H.; Fujiyoshi, Y. Crystal structures of the gastric proton pump. *Nature*.  **2018**, *556*, 214-218,‏ <https://doi.org/10.1038/s41586-018-0003-8>.

4. Huey, R.; Morris, G.M.; Forli, S. Using AutoDock 4 and AutoDock vina with AutoDockTools: a tutorial, The Scripps Research Institute Molecular Graphics Laboratory. **2012**, *10550*, 1000.‏

5. Yuan, S.; Chan, H. S.; Hu, Z. Using PyMOL as a platform for computational drug design. *Wiley Interdiscip. Rev. Comput Mol*. *Sci*. **2017**, *7*, e1298. <https://doi.org/10.1002/wcms.1298>.

6. Phillips, J. C.; Braun, R.; Wang, W.; Gumbart, J.; Tajkhorshid, E.; Villa, E.; Chipot, C.; Skeel, R. D.; Kalé, L.; Schulten, K. Scalable molecular dynamics with NAMD. *J. Comput. Chem*. **2005**, *26*, 1781-1802. <https://doi.org/10.1002/jcc.20289>.

7. Ribeiro, J. V.; Bernardi, R. C.; Rudack, T.; Schulten, K.; Tajkhorshid, E. QwikMD-Gateway for Easy Simulation with VMD and NAMD. *Biophys. J*. **2018**, *114*, 673a-674a,. https://doi.org/[10.1016/j.bpj.2017.11.3632](http://dx.doi.org/10.1016/j.bpj.2017.11.3632).

8. Humphrey, W.; Dalke, A.; Schulten, K. VMD: visual molecular dynamics. *J. Mol. Graph*.  **1996**, *14*, 33-38. <https://doi.org/10.1016/0263-7855(96)00018-5>.

9. Miller III, B. R.; McGee Jr, T. D.; Swails, J. M.; Homeyer, N.; Gohlke, H.; Roitberg, A. R. MMPBSA. py: an efficient program for end-state free energy calculations. *J. Chem. Theory Comput*. **2012**, *8*, 3314-3321. <https://doi.org/10.1021/ct300418h>.
